# PeNAC67-PeKAN2-PeSCL23 and B-class MADS-box transcription factors synergistically regulate the specialization process from petal to lip in *Phalaenopsis equestris*

**DOI:** 10.1186/s43897-023-00079-8

**Published:** 2024-04-23

**Authors:** Qingyu Xu, Zhenyu Yang, Yupeng Jia, Rui Wang, Qiyu Zhang, Ruonan Gai, Yiding Wu, Qingyong Yang, Guoren He, Ju Hua Wu, Feng Ming

**Affiliations:** 1 Development Centre of Plant Germplasm Resources, College of Life Sciences, Shanghai Normal University, Shanghai, 200234 China; 2 Shanghai Key Laboratory of Plant Molecular Sciences, College of Life Sciences, Shanghai Normal University, Shanghai, 200234 China; 3 National Key Laboratory of Crop Genetic Improvement, Hubei Hongshan Laboratory, Huazhong Agricultural University, Wuhan, China; 4 Hubei Key Laboratory of Agricultural Bioinformatics, College of Informatics, Huazhong Agricultural University, Wuhan, China

**Keywords:** *PeNAC67*, *PeSCL23*, *PeKAN2*, Lip development, ‘Perianth code’ model, *Phalaenopsis* orchids

## Abstract

The molecular basis of orchid flower development involves a specific regulatory program in which MADS-box transcription factors play a central role. The recent ‘perianth code’ model hypothesizes that two types of higher-order heterotetrameric complexes, namely SP complex and L complex, play pivotal roles in the orchid perianth organ formation. Therefore, we explored their roles and searched for other components of the regulatory network.

Through the combined analysis for transposase-accessible chromatin with high-throughput sequencing and RNA sequencing of the lip-like petal and lip from *Phalaenopsis equestris* var.trilip, transcription factor-(TF) genes involved in lip development were revealed. *PeNAC67* encoding a NAC-type TF and *PeSCL23* encoding a GRAS-type TF were differentially expressed between the lip-like petal and the lip. PeNAC67 interacted with and stabilized PeMADS3, which positively regulated the development of lip-like petal to lip. PeSCL23 and PeNAC67 competitively bound with PeKAN2 and positively regulated the development of lip-like petal to petal by affecting the level of PeMADS3. PeKAN2 as an important TF that interacts with PeMADS3 and PeMADS9 can promote lip development. These results extend the ‘perianth code’ model and shed light on the complex regulation of orchid flower development.

**Supplementary Information:**

The online version contains supplementary material available at 10.1186/s43897-023-00079-8.

## Core

Through the analysis with ATAC-seq and RNA-seq, we found that PeSCL23 and PeNAC67 competitively bound with PeKAN2 and positively regulated the development of lip-like petal by affecting the level of PeMADS3. PeKAN2 as an important TF that interacts with PeMADS3 and PeMADS9 can promote lip development in *Phalaenopsis equestris*.

## Gene & Accession Numbers

Sequence data from this article can be found in the database of the National Center for Biotechnology Information (NCBI) under the accession number: *PeNAC67*: XM_020733068.1, *PeSCL23*: XM_020733890.1, *PeKAN2*: XM_020727309.1, *PeMADS3*: XM_020715991.1, *PeMADS9*: XM_020737235.1, *PeMYB4*: XM_020729209.1, *PeMADS6A*: XM_020723641.1, *PeMADS2*: XM_020717890; *PeMADS10*: XM_020730780.1, *PeMADS6B*: XM_020740192.1, *PeMADS22*: XM_020719981.1.

## Introduction

Orchid is one of the most diverse and geographically widespread families of angiosperms. Their evolutionary success may be attributed to various factors, including epiphytism, exceptional adaptive capacity in different habitats, highly specialized pollination strategies, and diverse flower morphology (Aceto and Gaudio 2011; Cozzolino and Widmer 2005; Tremblay et al. 2005). The lip is a central organ in orchid pollination because of its strikingly distinct morphology and its direct opposition to the gynostemium. Its color patterns and structures are visual attractants, and it acts as a landing platform that guides pollinators towards the gynostemium (Lucibelli et al. 2021). Due to the central role of the lip in orchid reproduction, the developmental origin of the lip is a subject of intense study (Rudall and Bateman 2002; Endress 1994). Evolutionary and developmental biologists have shown great interest in exploring how orchid labella form their fascinating and complex structures.

The classic ‘ABC model’ has been proposed in the study of floral organ mutants of model plants *Arabidopsis thaliana* and *Antirrhinum majus*(Coen and Meyerowitz 1991), which laid an important foundation for the subsequent molecular regulation of floral organ development (Cozzolino and Widmer 2005). The discovery of D-class genes (*FLORAL BINDING PROTEIN 7* (*FBP7*), *FBP11* and *SEEDSTICK* (*STK*), *SHATTERPROOF1* (*SHP*1) and *SHP2*) and the identification of E-class genes (*SEPALLATA*) in *petunia* and *Arabidopsis* further extended the flower development model to the ‘ABCDE’ model. All of these floral sepal and petal homeotic genes encoded highly conserved B-class MADS-box transcription factors except *APETALA2* (*AP2*)(Theißen et al. 2016). C, D, E-class genes in orchids were also found to play an important role in perianth organ development including, but not limited to column and tepals formation (Li et al. 2022; Wang et al. 2020).

Except for ‘ABCDE model’ model (Tsai and Chen 2006), ‘orchid code’ (Mondragon-Palomino and Theissen 2008; Mondragon-Palomino and Theissen 2009), HOT model (Pan et al. 2011) and the ‘perianth (P) code’ model (Hsu et al. 2015) are widely recognized as the hypotheses to explain the identity characteristics of orchid perianth organs. According to the HOT model, the development of petal and lip are respectively controlled by the expression of clade-1 (*PeMADS2*-like genes) + clade-2 (*PeMADS5*-like genes) + clade-3 (*PeMADS3*-like genes) and clade-1 + clade-2 + clade-3 + clade-4 (*PeMADS2,5,3,4*-like genes) (Mondragon-Palomino and Theissen 2008), which all the genes belong to B-class MADS-box genes. Consequently, it is believed that the synergy among members of all B-class MADS-box genes is involved in the identification of orchid perianth, the growth of inflorescence and development of flower buds. In this process, *PI* and *AP3B* evolutionary branches determine the formation of sepals. *PI* and the combination of *AP3A1* and *AP3B* control the formation of lateral petals (Pan et al. 2011). *PI* and *AP3A2* evolutionary branch genes (and/or other *MADS*-box genes, such as *AGL6*-like, *SQUA*-like or unknown genes) control lip formation. This model also suggests that *PeMADS4* is a crucial gene for lip development in orchids (Pan et al. 2011).

The ‘P code’ model in *Phalaenopsis* emphasizes the partnership structure in the SP complex (OAP3–1-OAGL6–1-OAGL6–1-OPI as MADS2-MADS10-MADS10-MADS6 shown in *Phalaenopsis*) and the L complex (OAP3–2-OAGL6–2-OAGL6–2-OPI as MADS3-MADS9-MADS9-MADS6), which represents the two competitive complexes for forming sepals/petals or lips. When the SP complex is dominant, sepals and petals are formed; when the L complex is dominant, lips are formed (Hsu et al. 2015). In orchids, OAP3–1 orthologs are the main components of SP complexes, and a partnership with OAGL6–1 orthologs is established to determine the formation of sepals. In contrast, the OAP3–2 ortholog is the main component of the L complex, and a partnership with the OAGL6–2 ortholog is established to determine the formation of the lip. Further refinement of ‘P code’ indicates that two Orchidaceae B-class and *AGL6*-like genes may have evolved different functions in regulating tepal formation by forming two other complexes, including SP’ complex (OAP3–2-OAGL6–1-OAGL6–1-OP1) and the L’ complex (OAP3–1-OAGL6–2-OAGL6–2-OPI) (Hsu et al. 2021). Despite our limited comprehension of the intricate interactions between B-class *MADS*-box genes and various gene types in regulating the development of orchid lips, there is an imperative need for additional research to elucidate the specific roles of individual/class genes and enhance our comprehension of the gene regulatory network underlying orchid floral organ development.

Assay for transposase accessible chromatin with high throughput sequencing (ATAC-seq) is an innovative technique for studying epigenetic inheritance (Buenrostro et al. 2013). Closed chromatin would restrict the transcription factors to bind with promoters, resulting in gene silencing (Baylin and Schuebel 2007). ATAC-seq was also used to discover the regulatory mechanism in sweet osmanthus petals about their production of linalool and β-ionone (Han et al. 2022). In this study, we aimed to enrich and expand the ‘P code’ model by using ATAC-seq assay (Buenrostro et al. 2015) to reveal differentially expressed genes between the Lip and the lip-like petal (Pl) and confirmed that the newly identified TFs *PeNAC67* and *PeSCL23* were key regulators of the specialization process from petal to lip in orchids. Using molecular and genetic approaches, we found that PeNAC67 promotes the determination of lip identity, while PeSCL23 inhibits the lip formation. Based on RNA-seq, a key regulator of lateral floral organs, PeKAN2 encoded gene, were identified the association gene to connect *PeNAC67* or *PeSCL23* with *MADS3*. Both PeNAC67 and PeSCL23 can interact with PeKAN2; and PeNAC67 and PeKAN2 interacted with PeMADS3 to promote lip development. PeSCL23 competitively combines with PeKAN2 during this process. Taken together, these findings revealed the molecular mechanism by which PeSCL23-PeKAN2-PeNAC67-PeMADS3 regulates lip development of orchid flowers and provided a possibility of non-MADS transcription factors are involved in the regulation of perianth organ formation in *Phalaenopsis* orchids.

## Results

### ATAC-seq identified and predicted floral organ identity gene in *P. equestris* var.trilip

In the natural state, there is a mutant variety with *P. equestris* which was named as *P. equestris* var.trilip. *P. equestris* var.trilip are different in the structure of the petals with the original. Compared with *P. equestris*, *P. equestris* var.trilip’s second round of the two sides of the petals have a structure similar to the corpus callosum in the lip, and its overall shape is more similar to the lips(Li) which were regarded as lip-like petals(PL).

**Fig. 1:**
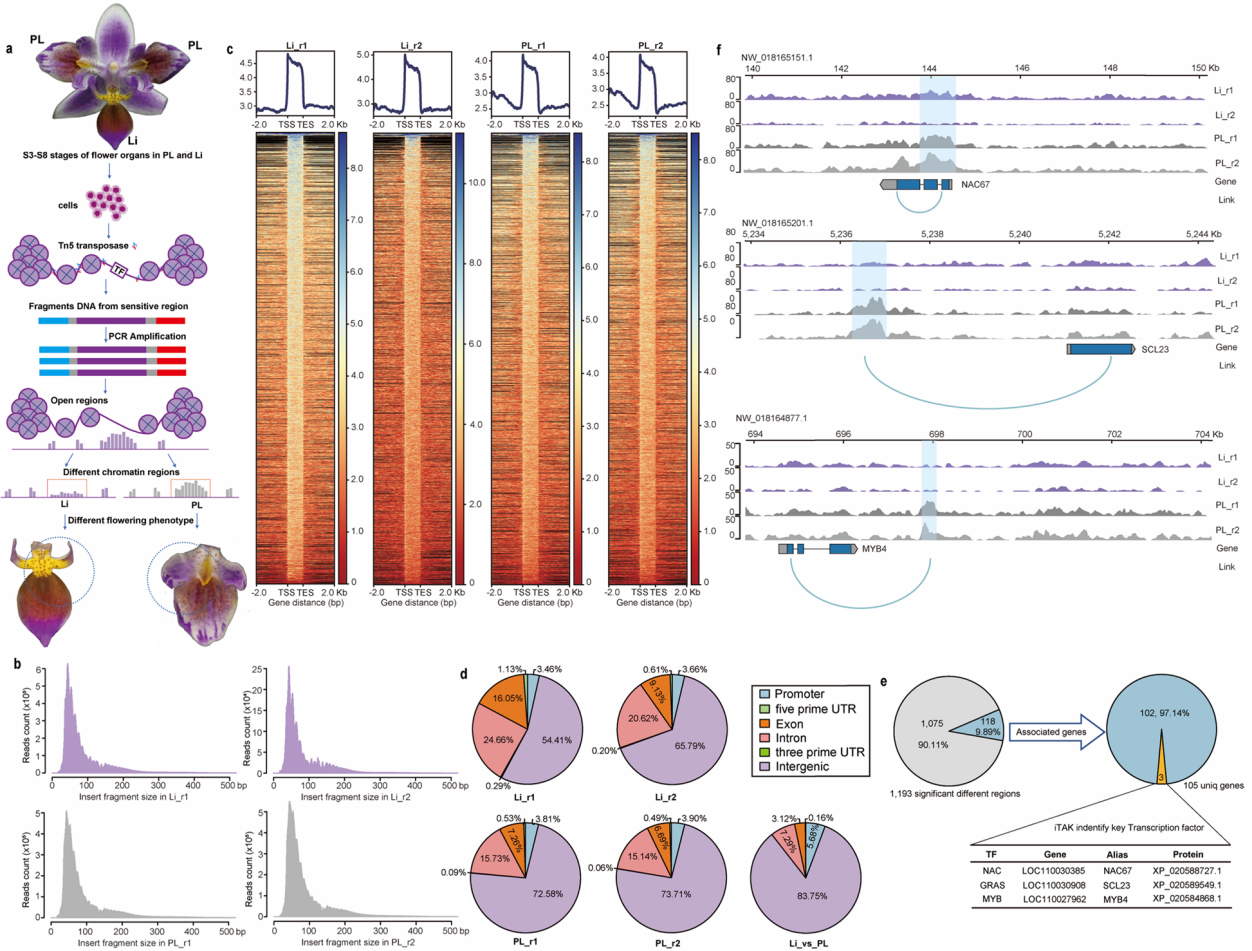
ATAC-seq is used to identify key transcription factors in lip development of *P. equestris* var.trilip*.* **a** Diagram of *P. equestris* var.trilip flower with petal (PL) and lip (Li) phenotypes, and method of using ATAC-seq to identify accessible regions in the genome of both mutants. **b** Distribution of insert fragment size in PL and Li samples. **c** Distribution of peaks within the 2-kb interval upstream and downstream of the transcription start site (TSS). **d** Proportions of genome regions covered by fragments generated in ATAC-seq analysis in PL samples, Li samples, and Li vs. PL. **e** Significantly different regions between PL and Li samples, and transcription factor (TF) genes among differentially expressed genes (DEGs). **f** Differences in transcription of three TF genes between PL and Li samples

We combined ATAC-seq and RNA-seq analysis to identify genes related to lip development in *P. equestris*. For the ATAC-seq analysis, we used *P. equestris* var.trilip samples with different flower phenotypes, including lip-like petals (PL) and lips (Li) (Fig. 1a and Fig. S2a). The distribution of peaks was found to be enriched within the 2-kb interval upstream and downstream of the transcription start site (TSS) (Fig. 1c). After aligning reads to the reference genome of Orchid Database V5.0, we counted the length of the inserted fragments and found that most were within 200-bp (Fig. 1b). Annotation of the sequences from the accessible regions of the genomes in the different samples revealed that most corresponding to intergenic regions, with a moderate number corresponding to introns and exons, and a few corresponding to promoters and 5′-untranslated regions (5′-UTRs) (Fig. 1d).

Next, sample repeatability heatmap of ATAC-seq datasets revealed the correlation between the two biological samples for *P. equestris* var.trilip in the ATAC-seq analysis (Fig. S1a). The lengths of differential peaks were in the range of 200–600 bp (Fig. S1b). A functional enrichment analysis of differential peaks associated genes revealed that many genes were associated with intrinsic membrane and integral membrane activities, as well as metabolism (Fig. S1c, d). Further analysis of these genes related to lip development revealed key transcription factors using the iTAK plant transcription factors database (Fig. 1e). Among them, three major TF genes (*PeSCL 23*, *PeNAC67* and *PeMYB4*) showed large differences in transcript levels between the Li and PL samples (Fig. 1f).

To validate the results from the ATAC-seq analysis, we conducted q-PCR analysis to detect the transcript levels of the TF genes identified by ATAC-seq, and found that the differences in transcript levels between samples were consistent with those detected from the ATAC-seq data (Fig. S2b). PeMYB4 was later verified as flower color regulator (Wang et al. 2022). Therefore, we speculated that PeSCL23 and PeNAC67 may be involved in lip formation. In a phylogenetic analysis of the differentially expressed genes (DEGs), PeNAC67 and PeSCL23 were found to be clustered with *Dendrobium catenatum* and *Apostasia shenzhenica*. Moreover, PeNAC67 and PeSCL23 have conserved domains in the NAC and GRAS families, as shown in Fig. S2.

### Co-expression of *PeNAC67* and *PeSCL23* with B-class *MADS-box* genes during flower development

**Fig. 2:**
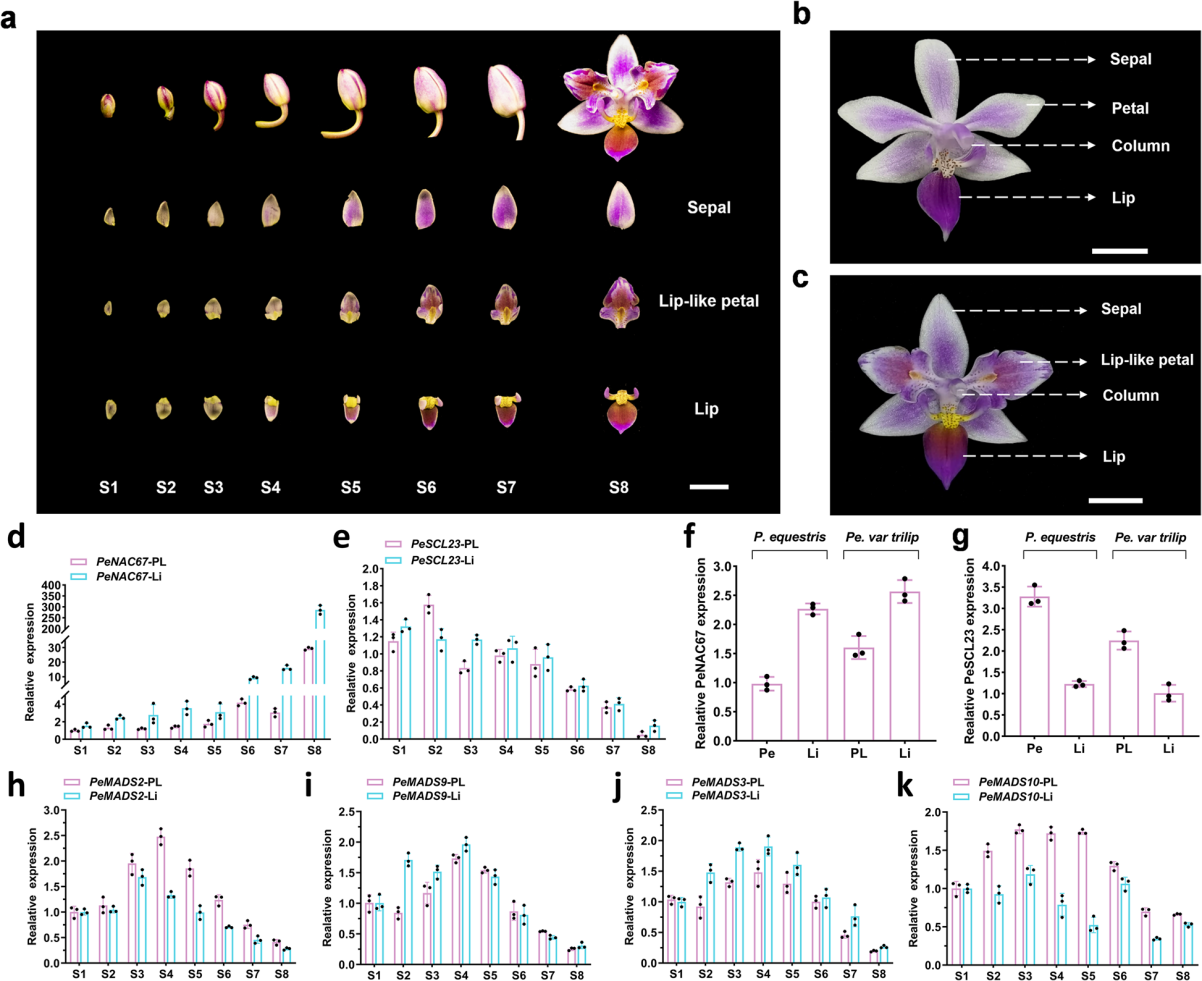
Expression of TF genes in *P. equestris* var.trilip and *P. equestris* flowers. **a** PL and Li from S3-S8 development flowers in the *P. equestris* var.trilip. Scale bar = 1 cm. **b** and (**c**) Different flower organs of *P. equestris* and *P. equestris* var.trilip. Scale bar = 1 cm. **d**, (**e**) and (**h**-**k**) Relative transcript levels of *PeNAC67* (**d**), *PeSCL23* (**e**), *PeMADS2* (**h**)*, PeMADS3* (**i**)*, PeMADS9* (**j**) and *PeMADS10* (**k**) in different organs of *P. equestris* var.trilip at different developmental stages. **f** and (**g**) Relative transcript levels of *PeNAC67* (**f**), *PeSCL23* (**g**) in the petal and lip of *P. equestris* and relative transcript levels in the lip-like petal and lip of *P. equestris* var.trilip at S1-S8 development stage. The expression patterns of all genes were determined using three replicates and were normalized using *PeActin4*. OAP3–1: PeMADS2, OAP3–2: PeMADS3, OAGL6–2: PeMADS9, OAGL6–1: PeMAD10

We detected the transcript profiles of *PeNAC67* and *PeSCL23* at eight different stages of flower development (Fig. 2a). The transcript levels of *PeNAC67* increased during flower development, with the transcript levels of *PeNAC67* peaking at the S8 stage (Fig. 2d). However, the transcript levels of *PeSCL23* decreased during flower development with higher transcript levels of *PeSCL23* during the S1-S5 stages (Fig. 2e). To explore the roles of *PeNAC67* and *PeSCL23* in lip formation, we measured their transcript levels in the petal and lip of *P. equestris* (Fig. 2b) and lip-like petal (PL), and lip of *P. equestris* var.trilip (Fig. 2c). The highest transcript level of *PeNAC67* was in the lip, followed by the lip-like petal, and its lowest transcript level was in the petal (Fig. 2f). In contrast, *PeSCL23* showed the opposite expression pattern, with the highest transcript levels in the petal and the lowest in the lip (Fig. 2g). Therefore, we speculated that lip formation may be positively regulated by *PeNAC67*, but negatively regulated by *PeSCL23*.

In addition, we detected the expression levels of the L complex (*PeMADS3*/*PeMADS9*) and the SP complex (*PeMADS2*/*PeMADS10*) at eight different stages of flower development of *P. equestris* var.trilip, and found the expression pattern of L complex genes (Fig. 2i, j) and SP complex genes were similar (Fig. 2h, k). However, the expression level of L complex genes in lip was higher than that of lip-like petals of S1-S8 flower development stages, while the expression level of SP complex genes in lip-like petals was higher than that of lip (Fig. 2h, k). These results demonstrated that L complex and SP complex genes expression were conserved in determining the identity of lip petal organs as previous study (Hsu et al. 2015).

### PeNAC67 correlating with MADS3 affected petal specialization in *P. equestris* var.trilip

**Fig. 3:**
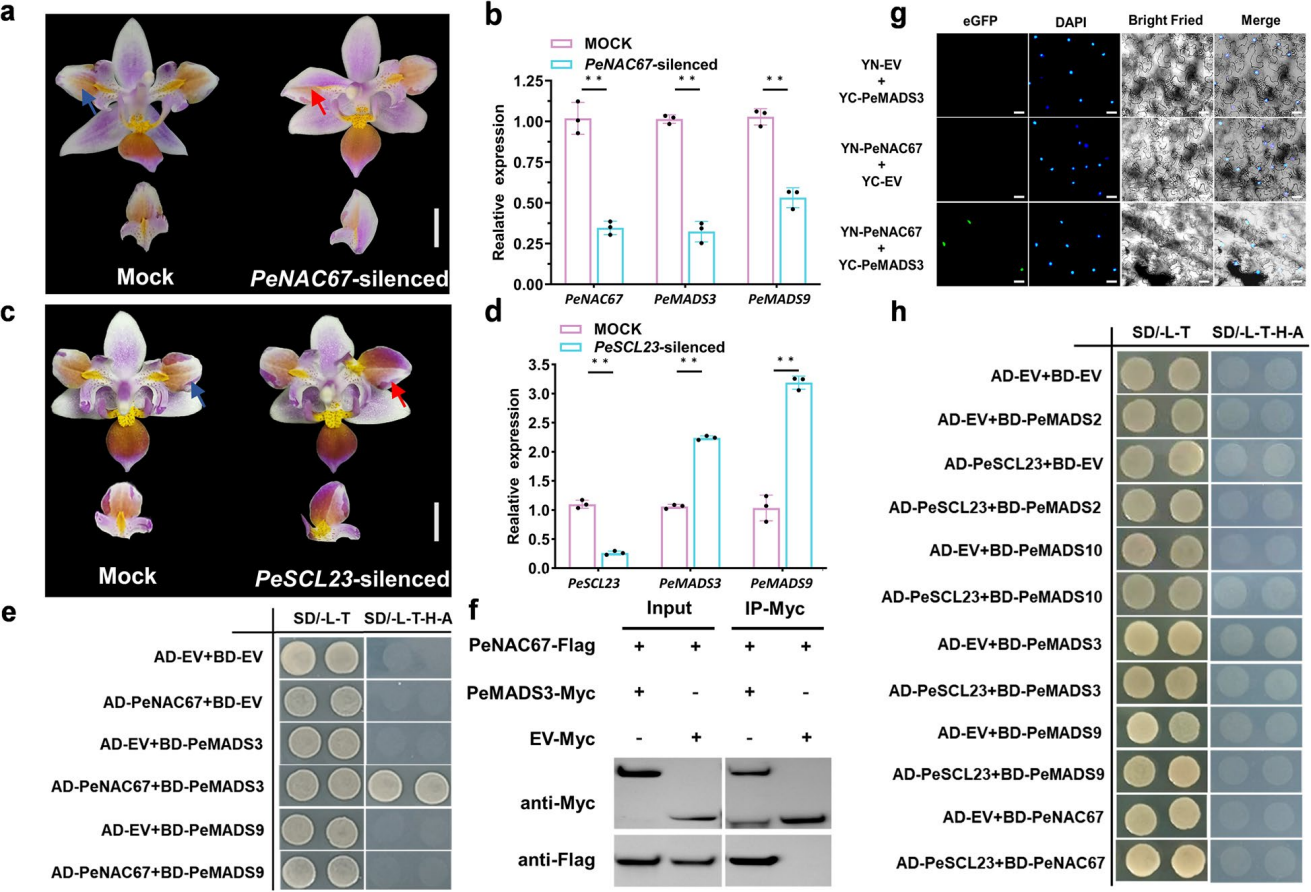
Phenotypes of *PeNAC67* and *PeSCL23* silencing lines and interaction between PeNAC67 with PeMADS3. **a** and (**c**) Mock and VIGS-treated lines with silenced expression of *PeNAC67* (**a**) and *PeSCL23* (**c**). The red arrows in (**a**) and (**c**) represent the part where the mutation occurs after VIGS treatment, and the blue arrows in (**a**) and (**c**) represent the corresponding part in the Mock. **b** and (**d**) Relative transcript levels of *PeNAC67*, *PeMADS3* and *PeMADS9* in Mock and VIGS-treated lines (**b**) and relative transcript levels of *PeSCL23*, *PeMADS3* and *PeMADS9* in Mock and VIGS-treated lines (**d**), asterisk in (**b**) and (**d**) indicated significant differences compared with the control, with one asterisk indicating *P* < 0.05 and two asterisks indicating *P* < 0.01. **e** Yeast two hybrid assay between PeNAC67 and PeMADS3/PeMADS9. **f** Co-IP was used to detect the interaction between PeNAC67-Flag and PeMADS3-Myc proteins transiently expressed in tobacco leaves. **g** Bimolecular fluorescence complementation (BiFC) assay was introduced to detect the interaction between PeNAC67 and PeMADS3 in tobacco leaves with DAPI staining. **h** Yeast two hybrid assay of PeSCL23 with B-class MADS-box protein

To explore the functions of *PeNAC67* and *PeSCL23* in lip development, we performed virus-induced gene silencing (VIGS) assays in *P. equestris* var.trilip. At 45 DPI, significant changes were observed in several floral phenotypes and morphological features. The *PeNAC67*-silenced lines showed normal vegetative growth and flowering time, but the lip-like petal was restored to the normal petal phenotype in almost 20% of the injected lines (Fig. 3a). The gene-silencing efficiency was assessed by q-PCR, which confirmed a significant down-regulation of *PeNAC67* in the silenced line (Fig. 3b). In the *PeSCL23*-silenced line, the lip-like petal was mutated into the lip phenotype (Fig. 3c), and q-PCR analysis confirmed the significant down-regulation of *PeSCL23* (Fig. 3d). Previous studies have shown that B-class and *AGL6* genes (*PeMADS3* and *PeMADS9* in *Phalaenopsis*) have a positive regulatory effect on the morphogenesis of orchid lips (Hsu et al. 2015, 2021). Therefore, we analyzed the transcript levels of *PeMADS3* and *PeMADS9* in the silenced lines and found that they both were significantly down-regulated in the *PeNAC67-*silenced lines (Fig. 3b), but up-regulated in the *PeSCL23*-silenced lines (Fig. 3d). Therefore, we speculated that *PeNAC67* and *PeSCL23* may positively and negatively regulate lip development, respectively, through *PeMADS3* and *PeMADS9*.

To explore the potential regulatory mechanism of PeNAC67 and PeSCL23 in floral development in *P. equestris*, we performed yeast-one-hybrid assay to screen the likely downstream targets of PeNAC67 and PeSCL23, and found that PeNAC67 and PeSCL23 (data not shown) can not directly interacts with the promoter of PeMADS3, PeMADS4, PeMADS6A, PeMADS6B, PeMADS22 and PeMADS9 (Fig. S3). Then we performed two-hybrid assays to test the interactions of PeNAC67 and PeSCL23 with PeMADS3, 9, only PeNAC67 was shown to interact directly with PeMADS3 (Fig. 3e). Next, the interaction between PeNAC67 and PeMADS3 were verified in *N. benthamiana* leaves through BiFC assay (Fig. 3g). To provide in vivo evidence, a strong interaction signal between PeNAC67 and PeMADS3 were revealed in Co-IP analysis (Fig. 3f). We also wondered whether PeSCL23 interacted with PeNAC67 or PeMADS3,9, but we did not detect any interactions among those proteins (Fig. 3h). These results indicated that PeNAC67 cooperates with PeMADS3 to regulate specialization process from petal to lip in *P. equestris*.

### PeKAN2 as the key factor in conjugate regulation between PeNAC67 and PeSCL23 during specialization process from petal to lip

**Fig. 4:**
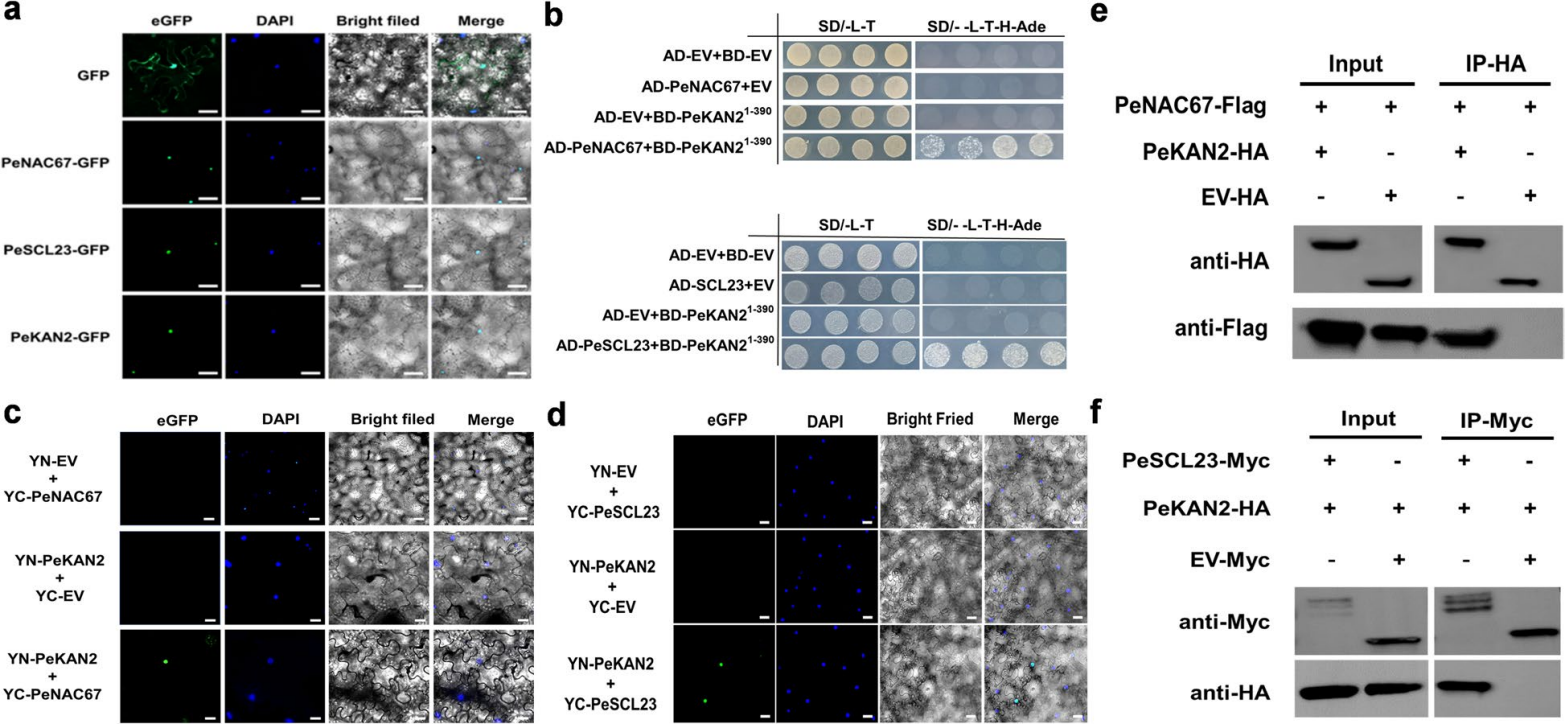
Analyses of interactions of PeNAC67 and PeSCL23 with PeKAN2. **a** Subcellular localization of PeNAC67-GFP, PeSCL23-GFP and PeKAN2-GFP proteins in *Nicotiana benthamiana* leaf epidermal cells with DAPI staining. Scale bars = 50 μm. **b** Yeast two hybrid assay of PeNAC67 and PeSCL23 with PeKAN2(1–390). **c** and (d) BiFC assay was introduced to detect the interaction PeNAC67 and PeSCL23 with PeKAN2 in tobacco leaves. **e** and (**f**) Co-IP was used to detect the interaction between PeNAC67-Flag and PeSCL23-Myc with PeKAN2-HA proteins transiently expressed in tobacco leaves

To explore the mechanism by which *PeSCL23* regulates petal morphogenesis, we performed RNA-seq analyses of the lip-like petal (PL) and lip (Li) in *P. equestris* var.trilip as well as petal (Pe) in *P. equestris* (Fig. S4a-d). To identify the candidate genes coding protein interacting with PeSCL23 or PeNAC67, yeast two-hybrid analysis was performed. Among them, only PeKAN2 was able to interact with PeSCL23 and PeNAC67, separately (Fig.S4e-f). Then PeKAN2 coding genes were chosen for later study. To study the subcellular localization of PeNAC67, PeSCL23 and PeKAN2, we fused their respective coding sequences with the green fluorescent protein (GFP) tag and introduced them into the leaves of *N. benthamiana*. The GFP signals of the PeNAC67-GFP, PeSCL23-GFP, and PeKAN2-GFP fusion proteins were in the nucleus of tobacco leaf epidermal cells (Fig. 4a), consistent with their putative functions as TFs in the nucleus. PeKAN2 was again confirmed to interact with both PeNAC67 and PeSCL23 by yeast two-hybrid assay. BD-PeKAN2^1–300^ could format interacting effector proteins with AD-PeNAC67 and AD-PeSCL23 (Fig. 4b). This result was confirmed in BiFC experiments (Fig. 4c, d) and Co-IP analyses (Fig. 4e, f). Thus, we speculated that the interaction of PeKAN2-PeNAC67, and PeKAN2-PeSCL23 may play important roles in the specialization process from petal to lip.

### Transcript level of *PeKAN2* was correlated with those of *PeMADS3/PeMADS9* and together regulate specialization process from petal to lip

**Fig. 5:**
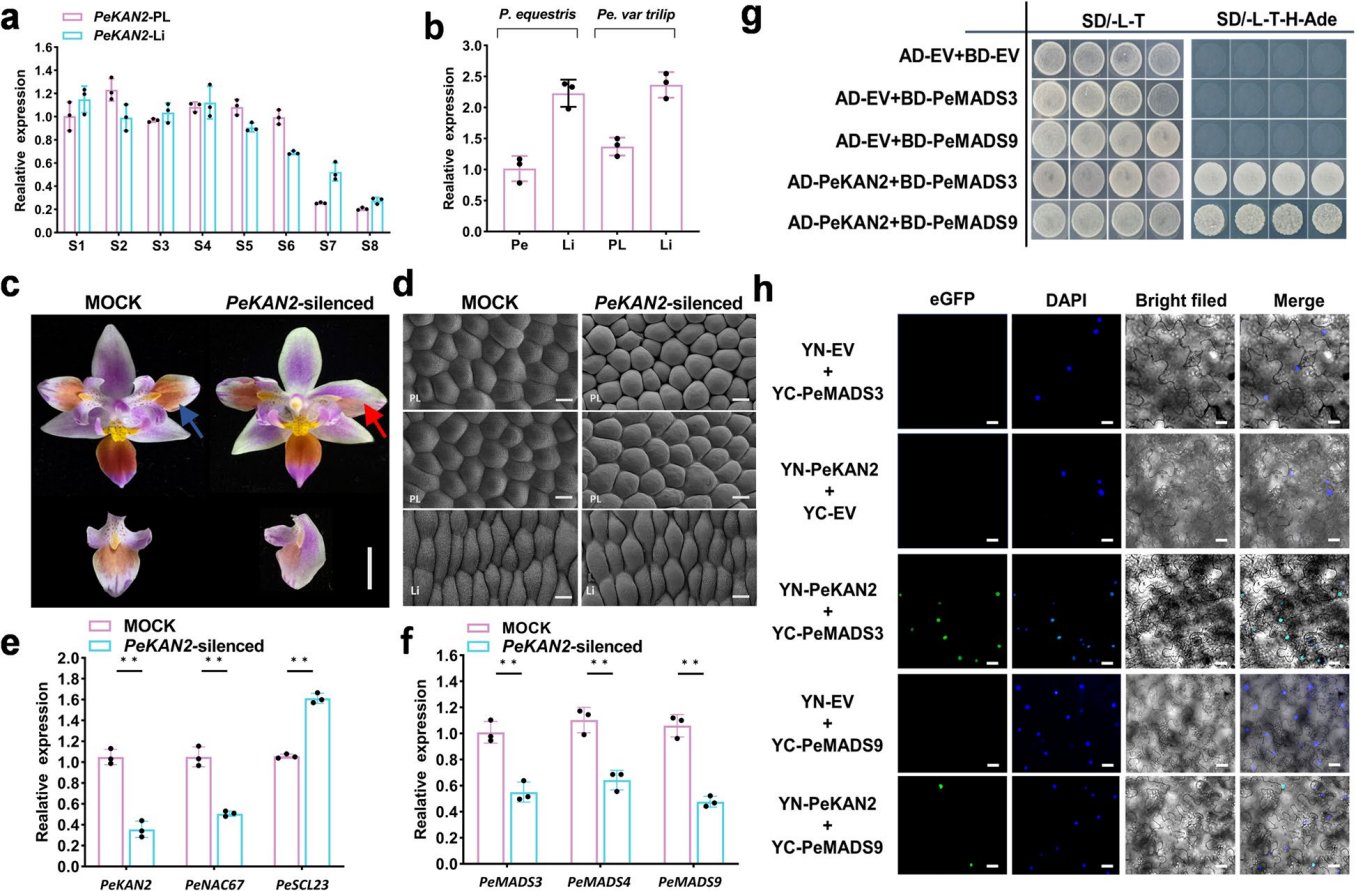
PeKAN2 expression during flower development and analyses of the role of PeKAN2. **a** Relative transcript levels of *PeKAN2* in different organs of *P. equestris* var.trilip at different developmental stages(from stages S1 to S8 development). **b** Relative transcript levels of *PeKAN2* in the petal and lip of *P. equestris* and relative transcript levels in the lip-like petal and lip of *P. equestris* var.trilip at S8 stage. **c** The phenotypes of *PeKAN2*-silenced lines. The red arrow represents the part where the mutation occurs after VIGS treatment, and the blue arrow represents the corresponding part in the Mock. **d** The epidermal cells of wild-type control (Left) and those of *PeKAN2*-silenced (right) flowers. Bar = 50 μm. **e** Relative transcript levels of *PeKAN2*, *PeNAC67* and *PeSCL23* in Mock and VIGS-treated lines. **f** Relative transcript levels of *PeMADS3*, *PeMADS4* and *PeMADS9* in Mock and VIGS-treated lines. **g** Yeast two hybrid assay of PeMADS3/PeMADS9 with PeKAN2. **h** BiFC assay was introduced to detect the interaction PeMADS3/PeMADS9 with PeKAN2 in tobacco leaves. Values are means ± SDs (*n* = 3). Asterisks in e and f indicate significant differences compared with the control, with one asterisk indicating P < 0.05 and two asterisks indicating P < 0.01

A high level of KAN2 expression was maintained during the development and maintenance of the lip (S1-S6), but decreased after the structure of the lip was basically formed in *P. equestris* var.trilip (S7-S8) (Fig. 5a). The highest levels of *PeKAN2* were observed in the lip (Li), followed by the lip-like petal (PL), and the lowest levels were observed in the petal (Pe) (Fig. 5b). To explore the function of *PeKAN2* in lip development, we performed a VIGS assay in *P. equestris* var. trilip. At 45 DPI after infection, the lip-like petal (PL) was restored to the petal (Pe) phenotype in the *PeKAN2*-silenced line (Fig. 5c). Scanning electron microscopy analyses of the petals of mock-infected and VIGS plants revealed that the lip-like (PL) petal showed different petal type (Fig. 5d). The epidermal cells of *P. equestris* var.trilip flowers (left panel) exhibited a flattened sepal/lip-like cell morphology of petal cells, whereas those of the *PeKAN2-*silenced line exhibited conical cell morphology of petal cells (Fig. 5d). The results of q-PCR analyses revealed significant down-regulation of *PeKAN2*, *PeMADS3/PeMADS9,* and *PeNAC67,* but significant up-regulation of *PeSCL23* in the *PeKAN2-*silenced line (Fig. 5e, f). To further explore the potential regulatory mechanism of *PeKAN2* in lip development, we performed yeast two-hybrid assays to confirm the interactions between PeKAN2 and PeMADS3/PeMADS9 (Fig. 5g). This interaction between PeKAN2 and PeMADS3/PeMADS9 was further confirmed in BiFC analyses (Fig. 5h).

### Interaction between PeKAN2 and PeMADS3 was enhanced by PeNAC67 but inhibited by PeSCL23

**Fig. 6:**
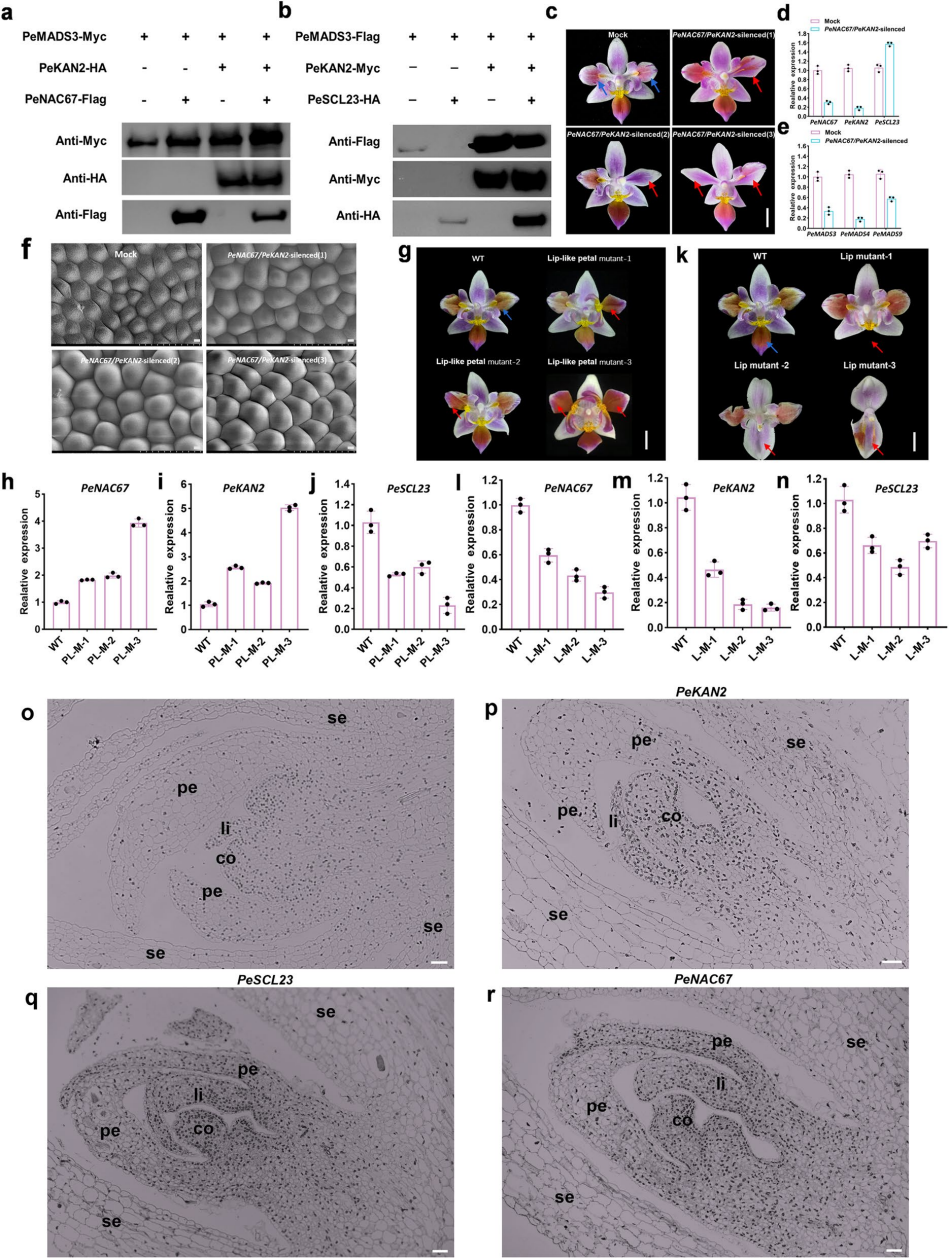
Biological function analysis of *PeNAC67*, *PeKAN2* and *PeSCL23* in *Phalaenopsis*. **a** The fusion constructs of PeNAC67-Flag, PeMADS3-Myc, PeKAN2-HA tags were transformed into *P.*Big chill in different combinations, and Myc/Flag/HA antibody was used for immunoprecipitation. **b** The fusion constructs of PeSCL23-HA, PeMADS3-Flag and PeKAN2-Myc tags were transformed into *P.*Big chill in different combinations, and Myc/Flag/HA antibody was used for immunoprecipitation. **c** Representative phenotypic analysis for three independent *PeNAC67* and *PeKAN2* double silencing lines are presented. The red arrow represents the part where the mutation occurs after *PeNAC67* and *PeKAN2* VIGS treatment, and the blue arrow represents the corresponding part in the Mock. **d** and (**e**) Relative transcript levels of *PeKAN2*, *PeNAC67* and *PeSCL23* in Mock and VIGS-treated lines (**d**) and relative transcript levels of *PeMADS3*, *PeMADS4* and *PeMADS9* in Mock and VIGS-treated lines (**e**). **f** The epidermal cells of wild-type control and those of *PeNAC67/PeKAN2* silenced flowers representative phenotypic analysis for three independent silencing lines was presented. Bar = 50 μm. **g** “Lip-like petal” mutant phenotype of *P. equestris* var.trilip*.* The red arrow represents the part where the mutation of “Lip-like petal”. **h**-**j** The expression of *PeNAC67*, *PeKAN2* and *PeSCL23* in “Lip-like petal” mutant phenotype of *P. equestris* var.trilip. (k) “Lip” mutant phenotype of *P. equestris* var.trilip. The red arrow represents the part where the mutation of “Lip petal”. **l**-**n** The expression of *PeNAC67*, *PeKAN2* and *PeSCL23* in lip mutant phenotype of *P. equestris* var.trilip. **o**-**r** In-situ localization of *PeKAN2* (**p**), *PeSCL23* (**q**) and *PeNAC67* (**r**) transcripts in *P. equestris* flower buds. Longitudinal sections were hybridized with DIG-labeled antisense. **o** A negative control was performed by sense probe. co, column; pe, petal; se, sepal; li, lip. Bar = 50 μm

To determine the biological functions of the four TFs (PeNAC67, PeSCL23, PeMADS3 and PeKAN2) in the regulation of lip development, we co-expressed their encoding genes in different combinations and performed western blot analysis to detect the expression of their encoded proteins. In the presence of PeKAN2, the protein contents of PeNAC67 and PeMADS3 were significantly increased, while in the presence of PeSCL23, the protein content of PeKAN2 was little changed but that of PeMADS3 was significantly decreased (Fig. 6a, b). In the *P. equestris* var.trilip, *PeNAC67,* and *PeKAN2* double-silenced lines, the lip-like petal was completely restored to a normal petal (Fig. 6c); and *PeNAC67*/*PeKAN2* was significantly down-regulated, while *PeSCL23*, which encodes a negative regulator of the lip, was up-regulated (Fig. 6d). Next, q-PCR analyses were conducted on genes encoding B-class and *AGL6* genes related to lip formation, revealing significant down-regulation of *MADS3/MADS4* and *MADS9* (Fig. 6e). Interestingly, *PeNAC67* and *PeKAN2* double-silenced lines caused the conversion of the lip-like petal into a petal structure (Fig. 6f). The epidermal cells of the control (mock) flowers petal exhibited conical cell morphology; lip exhibited a flattened cell morphology (Fig. 5d); lip-like petal (PL) exhibited an intermediate state of the lip and petal, whereas those of the double-silenced lines caused partial or total petal cell morphology (Fig. 5d, Fig. 6f). We also examined a mutant with true-lip petals (Fig. 6g) and found that *PeNAC67* and *PeKAN2* were significantly up-regulated, while *PeSCL23* was significantly down-regulated (Fig. 6h–j). Meanwhile, in several kinds of non-lip mutants (Fig. 6k), *PeNAC67* and *PeKAN2* were significantly down-regulated and *PeSCL23* was up-regulated (Fig. 6l–n). In situ hybridization showed that *PeKAN2* was concentrated at the base of the flower primordium (Fig. 6p). In situ results of *PeSCL23* and *PeNAC67* were consistent with *PeKAN2* (Fig. 6p-r). This suggests that these genes with co-expressed feature may play an important role in the flower organ formation of *P. equestris*. These findings provided further evidence that lip formation is positively regulated by PeNAC67/PeKAN2 and negatively regulated by PeSCL23. Together, these results showed that PeNAC67/PeKAN2 promotes lip development by interacting with PeMADS3 and that PeSCL23 inhibits lip development in *P. equestris* var.trilip by competitively interacting with PeMADS3.

## Discussion

### The first successful application of ATAC-Seq to identify important genes in a horticultural flower

Identification of TF binding sites is a crucial step in understanding the function of TFs and regulatory networks in organisms. ATAC-seq is a simple protocol to detect open chromatin, a powerful tool to explore protein-DNA interactions (Chen et al. 2021). The binding of TFs to *cis*-elements is often associated with accessible chromatin regions (Lu et al. 2017). Therefore, identifying these regions across the genome can enhance our understanding of the relationship between TF binding, chromatin status, and the regulation of gene expression. In previous studies, ATAC-seq has been utilized to analyze the differences in chromatin accessibility and the TF regulatory network between stem cells in the *Arabidopsis* shoot apical meristem and differentiated mesophytic cells (Bajic et al. 2018), and to reveal different types of accessible chromatin associated with H3K27me3 and DNA methylation in *A. thaliana* (Zhang et al., 2020). ATAC-seq was used to explore root-specific chromatin accessibility in *Arabidopsis*, and revealed that gene-distant sites are enriched with binding motifs of TFs essential for root development. This finding suggest that factors involved in defining organ identity may function via long-range chromatin interactions (Tannenbaum et al. 2018). While ATAC-seq has been extensively used for the systematic identification of *cis*-regulatory regions in plant genomes, it has rarely been used for horticultural plants. In our study, we used ATAC-seq analyses to explore the role of a NAC-type TF (PeNAC67) and a GRAS-type TF (PeSCL23) in regulating lip development in *P. equestris* flowers (Fig. 1). Our findings shed light on the transcriptional regulation of lip development in orchids and demonstrate the potential of ATAC-seq to provide new information about gene regulation in horticultural plants.

### New function of non-MADS genes in flower organ development

Floral homeotic genes encoding MADS-box TFs play important roles in flower development. Such genes include *AGAMOUS-LIKE6* (*AGL6*)-like MADS-box genes, which are crucial for lip development (Hsu et al. 2015). Recent studies have identified non-MADS-box genes that play roles in the development of reproductive organ development in *Phalaenopsis* orchids, including two *TEOSINTE-BRANCHED*/*CYCLOIDEA*/*PCF* genes (*PePCF10* and *PeCIN8*) and two *DROOPING LEAF*/*CRABS CLAW* genes (*PeDL1* and *PeDL2*) (Lin et al. 2016; Chen et al. 2021). Here, we used bioinformatics methods to identify three TFs (NAC67, SCL23, KAN2) that are involved in the process of petal specialization or lip morphogenesis (Fig. 3 and Fig. 5).

The GRAS gene family (named after the first three identified members, GAI, RGA, and SCR) encodes TFs involved in plant growth and development (Liu et al. 2019). REPRESSOR OF GA (RGA), GA INSENSITIVE (GAI), RGA-LIKE1 (RGL1), RGL2, and RGL3, are all DELLA proteins (Sun et al., 2004). The RGA TFs inhibit gibberellin signaling and induce vegetative growth and flowering initiation; and RGL1 and RGL2 also regulate flower development (Silverstone et al. 1998; Griffiths et al. 2006; Zentella et al. 2007). The SCARECROW (SCR) and SHORT-ROOT (SHR) TFs are involved in bundle sheath cell and leaf development (Lee et al. 2008). Our findings demonstrate the regulation of floral organ development by members of the GRAS gene family in *Phalaenopsis* orchids (Fig. 3; Fig. 6).

Previous studies have shown that NAC (NAM, ATAF1/2, CUC1/2) TFs participate in plant growth and development, biological and abiotic stress responses, and the regulation of flower development (Ernst et al. 2004; Vroemen et al. 2003). Mutations in *CUC1/2* cause defects in the separation of cotyledons (embryonic organs), sepals, and stamens (floral organs) (Aida et al., 1997), and mutations in *FveCUC2a* result in the growth of leaves with smooth margins (Zheng et al., 2019). *Arabidopsis* lines overexpressing NAC092 have a significantly reduced number of pollen grains (Balazadeh et al. 2010). In rose, ethylene regulates *RhNAC100*, which encodes a NAC TF that inhibits the expansion of petal cells and significantly reduces petal size (Pei et al. 2013). In tomato, miR164-regulated *NAM* genes play key roles in floral-boundary specification (Hendelman et al. 2013). CUC1 and CUC2 are involved in the formation of the carpel margin in the developing meristem of *Arabidopsis* (Kamiuchi et al. 2014). Our results demonstrate the role of PeNAC67 in the orchid petal specification process (Fig. 3; Fig. 6).

The development of lateral organs is an important part of morphogenesis in higher plants (Zhang et al. 2019; Wang et al. 2021a, 2021b). The TFs that control the development of lateral organs, such as YABBY and KANADI, control the formation of polarity in developing lateral organs of *Arabidopsis* (Du et al. 2018). Loss of function of *KANADI* genes (including *KAN1, KAN2, KAN3,* and *KAN4*) led to differences in cell morphology, cell number, and other traits on the abaxial surface of lateral organs (Eshed et al. 2001). *KAN1–3* are expressed specifically in the phloem in the vascular tissue during late leaf development, and also on the dorsal side of floral organ primordia during reproductive development (Eshed et al. 2004). KAN acts as a transcriptional repressor during the formation of the dorsoventral region of plant leaves. It inhibits the expression of *AS2* encoding the adaxial regulator ASYMMETRIC LEAVES2 (AS2) in adaxial cells to promote the establishment of the adaxial identity of lateral organs and the adaxial recognition of leaves and carpels (Kerstetter et al. 2001; Eshed et al. 2001; McAbee et al. 2006). The AS2 protein complex plays a central role in antagonistic interactions among polar specification genes in *Arabidopsis* leaves. Interestingly, stamens, carpels, and possibly some highly specialized petals and leaves do not remain as lamellar structures during development, but undergo a dramatic change in adaxial to abaxial polarity (Fukushima and Hasebe 2014; Toriba et al. 2010; Yao et al. 2019). Moreover, ventral refinement of complex petals is associated with changes in the expression domains of adaxial and/or distal genes, and the ventral refinement of petals and leaves is conserved (Fu et al. 2022). Thus, members of the *KANADI* family may play an important role in the establishment of floral organ boundaries. We found that *PeKAN2* could function to promote the the formation of lip (Fig. 5). And the function may be achieved by interacting with MADS genes (Fig. 5g).

### PeKAN2 regulated lip development by serving as a bridge between PeNAC67 and PeSCL23

**Fig. 7:**
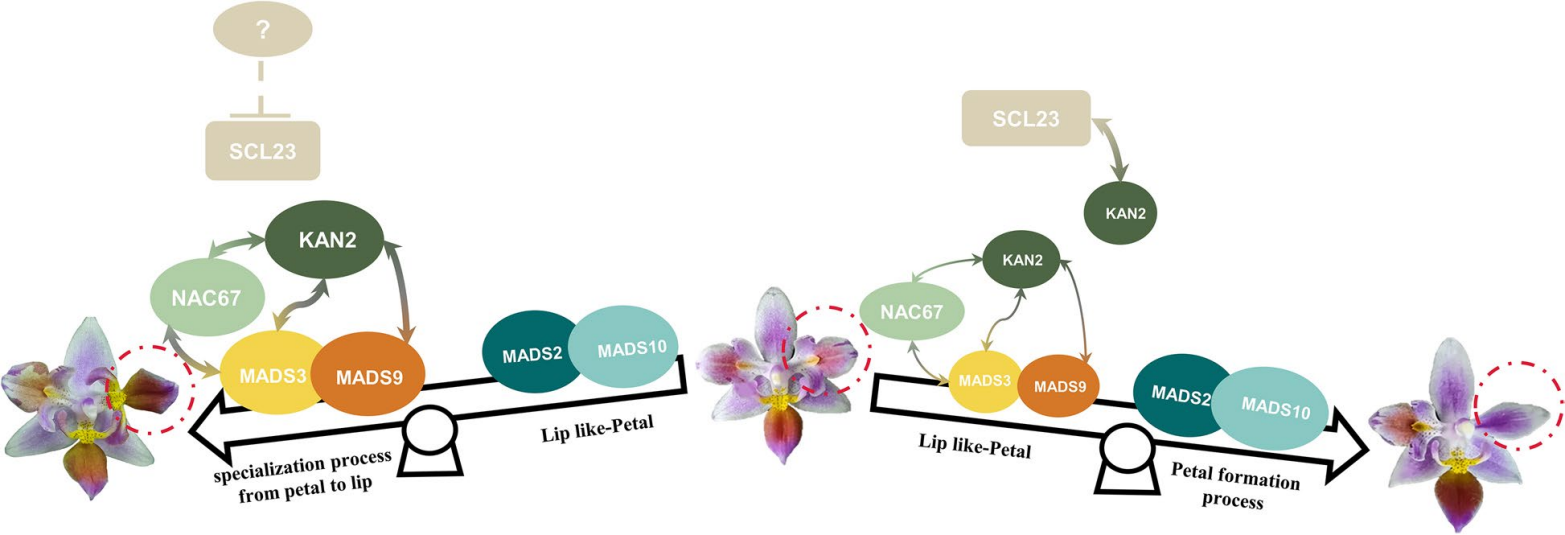
Model of PeNAC67, PeSCL23, PeKAN2 cooperation with MADS box protein to regulate the lip development in *P. equestris* var.trilip. During the specialization process from petal to lip, PeSCL23 translation was inhibited, PeNAC67 interacted with PeKAN2, and enhanced the stability of *PeMADS3*; Meanwhile PeKAN2 also could correlating with PeMADS9. The development of the lip was promoted through enhancing PeMADS3 activity. During the formation process of petals, PeSCL23 and PeNAC67 competitively interacted with PeKAN2, which brought about the decreasing of the PeMADS3 activity, and instead promoting petal formation

The development of flowers is crucial for the reproduction and continuation of angiosperms. In this study, we identified a NAC family TF PeNAC67 in *P. equestris* (Fig. 2) and showed that PeNAC67 promotes the formation of the lip (Fig. 3a, 6c). MADS-box genes play key regulatory roles in the flower development of plants (Hsu et al. 2015). q-PCR analysis of gene transcript profiles in the *PeNAC67*-slience strains revealed that MADS-box family genes controlling the formation of the lip were down-regulated, consistent with the phenotype (Figs. 3b, 6e). Another important finding of this study is that PeKAN2 positively regulates lip formation (Fig. 5) and interacts antagonistically with PeSCL23 during lip formation in *Phalaenopsis* (Fig. 6). PeSCL23 may inhibit PeKAN2 by interacting with PeMADS3, while PeNAC67 may enhance the activity of PeKAN2 by interacting with PeMADS3 (Fig. 6a, b). We conclude that PeKAN2 and PeNAC67 are involved in the ‘P-code’ model by directly interacting with PeMADS3 and enhancing the regulatory actions of PeMADS3 and PeMADS9 in labial conversion. PeSCL23 indirectly impairs the functions of PeMADS3 and PeMADS9 by interacting with PeKAN2 (Fig. 7). In this way, PeKAN2 functions as a bridge between PeNAC67 and PeSCL23 to regulate lip development. This bridging function is worthy of further study. In addition, MADS-box genes play an important role in both the classical ‘ABCDE’ floral organ development model and the ‘P-code’ model of Orchidaceae (Theißen et al. 2016; Hsu et al. 2015). Little is known about the regulation of MADS-box genes by non-MADS gene families (Thomson and Wellmer 2019; Sharma et al. 2017). The results of the present study confirm the interaction between PeKAN2 and PeMADS3/PeMADS9(Fig. 5). Although the site of their interaction is still unclear, given the conservation of MADS-box family members (Gramzow and Theissen 2010; Theißen et al. 2016; Lai et al. 2019), PeKAN2 is likely to interact with other MADS-box proteins. Our results showed that non-MADS-box genes are also involved in the regulation of floral organ development via MADS-box genes.

Using VIGS to silence *PeKAN2* resulted in a petal-to-lip conversion phenotype, in which petal epidermal cells were transformed from flattened to conical (Fig. 5d). KAN1 and KAN2 are involved in the establishment of polarity in most lateral organs, including leaf and floral organs such as sepals, petals, stamens, and carpels (Eshed et al. 2001; Zheng et al. 2018), and KANADI and TFs such as YABBY and ARF3/4 are involved in the dorsal-ventral formation of plant leaves by controlling the development of the distal plane and the axial plane (Kumaran et al. 2002; Fukushima and Hasebe 2014). On the basis of our results, we suggest that PeKAN2 regulates the petal-to-lip conversion through the establishment of polarity during floral organ development (Fig. 5c, d). The lip of orchids is derived from petal specialization. Further elucidation of the PeKAN2 mechanism may reveal how orchids are evolutionarily different from other plants.

## Methods

### Plant materials

The wild-type *P. equestris* and its peloric mutant *P. equestris* var.trilip (with distinct lateral petals have been transformed into lip-like petals) used in this study were purchased from Ruifeng Horticulture (Changhua, Taiwan). The lip-like petal and lip of the peloric mutant were used for ATAC-seq and virus-induced gene silencing (VIGS) experiment. Both *P. equestris* and its peloric mutant were used for RNA-seq analysis, gene isolation and cloning, spatial and temporal genes expression by using quantitative real-time PCR (q-PCR). Since a single lip sample could not achieve the amount required for sequencing, the samples used for sequencing in each period were taken from those multiple flowers at the same stage. Meanwhile, we treated *P. equestris* var.trilip by 40 Gy ^60^Co γ-irradiation and achieved lip-like petal mutant (PL-M) and lip mutant (L-M) for q-PCR. *Phalaenopsis*. Big Chili(a commercial cultivar with big red flowers)were collected from Taida Horticultural Co. and used for western blot experiment. All plants were kept in the greenhouse at Shanghai Normal University (SHNU) with a controlled temperature of 27/22 °C (day/night). *Nicotiana tabacum* L. cv plants were grown in growth chamber maintained at 25/20 °C with a 12/12 light-dark photoperiod.

### ATAC-seq

DNA was extracted from lip (Li) and lip-like petal (PL) of *P. equestris* var.trilip (samples combined from S3 to S8 developmental stages) (Fig. 1a). Every After detecting the quality of DNA extraction, equimolar amounts of DNA from different tissues were collected and mixed for high-throughput sequencing. ATAC-seq on Illumina HiSeqTM 2000 with 150-bp paired-end reads. Quality control processing was performed using the fastqc software (version 0.11.5) on the raw data. After quality control evaluation, the raw data were filtered using Trimmomatic software (version 0.36) with default parameters, and the filtered data were again processed by the software fastqc (version 0.11.5) for quality control to ensure high-quality clean data.

The clean reads were aligned to *P. equestris* reference genome in Orchid Base V5.0 using Hista2 (version 2.0.1-beta). Multi-mapped reads were removed by using sambamba (version 0.7.1) and then PCR duplicated reads were removed using the default parameters of Picard (version 2.16.0). Peaks were identified using MACS2 (version 2.1.2) with parameters “--nomodel-q0.05 --extsize 200--shift -100-g 1.06e9 --keep-dup all-B --call-summit”. These peaks were annotated in R package ChIPseeker and 1500 bp upstream from the transcriptional start site (TSS) as putative promoter regions.

Based on the peaks detected above, we used the R package Diffbind to identify differential peaks from multiple experiments. After detecting all the differential peaks, the significantly differential peaks with |logFC| > 1 & FDR < 0.05 threshold were identified. Then, the differential peaks were annotated using ChIPseeker and the nearest genes around these differential peaks were used to identify plant TFs, as well as gene ontology enrichment analysis. All the raw SRA had uploaded to the National Center for Biotechnology Information (NCBI) database: ATAC-seq of *P. equestris* and *P. equestris* var. *triple* (SRA: SRP407906, BioProject: PRJNA899518).

### RNA-Seq

RNA was extracted from lip (Li) and lip-like petal (PL) of *P. equestris* var.trilip (sample combined from S3 to S8 developmental stages) and from petal (Pe) of *P. equestris* (sample combined from S3 to S8 developmental stages). The RNA-seq transcriptome libraries were prepared using an Illumina TruSeq™ RNA Sample Preparation Kit (San Diego, CA, USA). RNA-Seq libraries were sequenced in a single lane on an Illumina NovaSeq 6000 sequencer (Illumina, San Diego, CA, USA) for 2 × 150 bp paired-end reads. The high-quality clean reads (Table S2) were compared with the reference genome of *P. equestris* to obtain mapped reads for subsequent transcript assembly and gene expression calculations. The data quality of the transcriptome was assessed (Table S3). Based on the selected reference genome sequence, mapped reads were assembled using StringTie or Cufflinks software and compared with the original genome annotation information form Orchid Base V5.0(Table S4) to find original unannotated transcript regions and discover new transcripts and genes in this species, thereby complementing the original genome annotation information. We performed a Venn diagram analysis of the obtained data to identify 28 TFs commonly expressed in Pe, PL and Li samples (Fig. S3a), and then classified them into their different families (Fig. S3b). From these 28 differentially expressed TFs, we screened the KANADI2 (encoded by *PeKAN2*) for subsequent experimental analysis (Fig. S3c).

### Sequence alignments and phylogenetic analysis

The sequences of *NAC* and *SCL* gene families from other plants were downloaded from the National Centre for Biotechnology Information (NCBI) (http://www.ncbi.nlm.nih.gov) for phylogenetic analysis. Multiple sequence alignments of NAC and SCL proteins sequences using ClustalW (http://www.clustal.or2ag/clustal2/), and phylogenetic analysis was performed using the Maximum Likelihood (ML) method with MEGA(v10)(https://www.megasoftware.net/) (Kumar et al., 2018). Bootstrap values were calculated with 1000 replicates. The protein accession numbers are listed in Supplementary Table S1.

### RNA isolation and quantitative real-time RT-PCR (q-PCR)

Total RNA was treated with DNase (NEB, Hertfordshire, UK) to remove remnant DNA. First-strand cDNA was synthesized using the Superscript III kit followed manufacture’s instruction (Invitrogen, CA, USA). The quantitative real-time PCR was performed using SYBR GREEN PCR Master Mix (Applied Biosystems, Warrington, UK) on ABI 7500, Applied Biosystems System. The PCR was performed with the following reaction conditions: 95 °C for 10 min, 40 cycles of 95 °C for 15 s and 60 °C for 1 min. For real-time q-PCR, each gene was analyzed in biological triplicates. *PeActin4* (PACT4, AY134752) of *Phalaenopsis* were recruited as an internal control (Cai et al. 2015), and data analysis was performed using the Sequencing Detection System v1.2.3 (Applied Biosystems). All the primers used in this study are listed in Supplementary Table S1.

### Subcellular localization

The open reading frames (ORFs) of PeNAC67, PeSCL23 and PeKAN2 were cloned into pCAMBIA1300 vector to create 35S:PeNAC67-GFP, 35S:PeSCL23-GFP and 35S:PeKAN2-GFP constructs using the pEASY®-Basic Seamless Cloning and Assembly Kit (Transgen Biotech, China). The procedure used for *Nicotiana benthamiana* subcellular localization was described in a previous study (Wang et al. 2022). The primers were listed in Table S1.

### Virus-induced gene silencing experiment(VIGS)

We followed the protocol described in (http://www.bio-protocol.org/e1359) for the VIGS experiment in orchids. The AttB site is used for in vitro recombination with the attP site in the VIGS vector pCymMV (kindly provided by Dr.HH Yeh, Agricultural Biotechnology Research Center, Academia Sinica.)to generate recombinant clones using Gateway BP Clonase II Enzyme Mix (Invitrogen). The pCymMV-*PeNAC67*, pCymMV-*PeSCL23* and pCymMV-*PeKAN2*, as well as the empty vector pCymMV (as a control group), were transformed into *Agrobacterium tumefaciens* EHA105 for further inoculation. For *P. equestris* var.trilip leaf infiltration, we injected the suspension into the leaves of 6–9 plants for each pCymMV-Gateway construct directly below where the inflorescence emerged. Then we analyzed 18–27 flowers samples at 45 DPI (days post inoculation). Experiments were repeated 3 times independently. The primers used are listed in Supplementary Table S1.

### Yeast two-hybrid assay

The full-length coding sequences of *PeNAC67*, *PeSCL23*, *PeKAN2* and B-class MADS-box genes were cloned and inserted into pGADT7 (bait) or pGBKT7 (prey) vectors (Niu et al., 2015) using the pEASY-Basic Seamless Cloning and Assembly Kit (Transgen Biotech, China). SD/−Leu-Trp was used as a common medium, whether SD/−Leu-Trp-His-Ade was used as a screening medium. The sequences of the primers used for amplification are shown in Supplemental Table S1.

### Bimolecular fluorescence complementarity(BiFC)experiment analysis

The full-length coding sequences of *PeNAC67, PeSCL23, PeKAN2, PeMADS3*, and *PeMADS9* genes were fused to either the N−/C-terminus of yellow fluorescence protein (YFP, nYFP/cYFP). The empty vectors including nYFP or cYFP were used as negative controls. *N. benthamiana* leaves were collected 3 days post infection and stained with 150 μg/ml DAPI (Sigma, USA) and observed under Olympus FV3000 confocal scanning microscope. YFP and DAPI fluorescence were observed at excitation wavelengths of 505 nm and 340 nm, respectively. The primers used in the BiFC are listed in Supplemental Table S1.

### *In stiu* hybridation

RNA in situ hybridization was performed to investigate the expression pattern of PeKAN2, PeSCL23, and PeNAC67 as described (Komminoth 1992). The lips from wild type plants at S1 were fixed in FAA (50% ethanol, 5% acetic acid and 3.7% formaldehyde). Paraffin embedded samples were sectioned with a sliding microtome (Leica, Germany), dewaxed, and then digested with Proteinase K (Roche, Switzerland). The dehydrated slides were hybridized with corresponding probes and incubated with anti-digoxigenin-AP Fab fragments. After washing, the signals were detected with the DAB stock solution (Roche, Switzerland). The probes labeled with digoxigenin were synthesized by Shanghai Gefan Biotechnology Co. Ltd. and the sequence were listed. KAN2: 5′-CGTAGAAAGGTGAGATCTTGGTGATGGGATTCATTGAGTAAAGGA-3′, NAC67: 5′-CTGTGAAGAGTTTCAGCAAGTCTATACTCGTGCATGATCCAGTTA-3′, SCL23: 5′-CTTCGAGGAGATCGGAATCAGGGCCAATTCCGGTGATTCGAATTG-3′. Sense probe: 5′-UUGUACUACACAAAAGUACUG-3′.

### Co-immunoprecipitation (Co-IP) assay

*PeNAC67* and *PeMADS3* genes were cloned into PEG104 vectors with a Flag tag, while *PeSCL23, PeKAN2*, and *PeMADS3* genes were cloned into PEG104 vectors with a Myc tag, and *PeSCL23* and *PeKAN2* genes were cloned into PEG104 vectors with a HA tag, using Gateway™ LR Clonase™ II Enzyme mix (Invitrogen, USA) with primers listed in Supplementary Table S1. *A. tumefaciens* cells containing the various constructs were collected and suspended at OD_600_ = 1.0 as described above. Infiltration of the *A.tumefaciens* were performed into the leaves of 4-week-old *N. benthamiana* plants, harvested 2 days post incubation, and snap-frozen and ground to powder. Proteins were extracted with extract buffer and then incubated at 4 °C for 4 h in the presence of monoclonal anti-Myc or anti-HA antibody-conjugated beads. Protein extracts were separated on 8% SDS-PAGE gels, and then transferred to polyvinylidene difluoride membranes using transferring buffer. The membranes were then blocked with skimmed milk for 1 h at room temperature. The target proteins were incubated with anti-FLAG, anti-Myc or anti-HA (1:5000 MBLbio, China) at room temperature for 1 h, and sequentially incubated with secondary peroxidase-conjugated anti-mouse antibody (MBLbio, China) at room temperature for 1 h, and observed by ChemiScope series (Clinx Science instruments Co., Ltd.).

### Transient overexpression in *P.* Big chili and Western blot

For the transient assay in *P*. Big Chili petal, *A. tumefaciens* cells containing various constructs were collected and suspended at OD_600_ = 1.0 as described above. Combination of *A. tumefaciens* with different tag genes were injected into different positions of the same petal with a 1 ml medical syringe. Four groups were injected into each petal and each treatment contained experimental triplicates. The infiltrated *P.* Big Chili petals were kept in the dark at 22 °C for 1 days, followed by growth at 18 °C under a 16 h/8 h light/dark cycle for 4 days. Five petal discs from each group were frozen in liquid nitrogen and ground to powder. The western blot was performed as mentioned in Co-IP.

### Cryo-scanning electron microscopy

The perianths were dissected, frozen using liquid nitrogen, and then transferred to the sample preparation chamber at − 197 °C. The samples were sublimated for 2 min at − 95 °C and were observed under a cryo-scanning electron microscope (Hitachi S4800) after gold coating.

### Supplementary Information

**Additional file 1: Fig. S1**. Characteristics and functional analysis of differential peak and related gene. (a) Sample repeatability heatmap of ATAC-seq datasets. (b) Distribution of peak sizes of sequencing samples. (c) Gene Ontology annotations of differential peak-related genes. GO enrichment include three parts: Molecular Function (MF), Biological Process (BP) and Cell Component (CC). (d) KEGG pathways analysis of differential peak-related genes.

**Additional file 2: Fig. S2.** Sequence characteristics of PeNAC67 and PeSCL23. (a) Phenotypic analysis of *P. equestris* var.trilip. Se: sepal, PL: lip-like petal, Li: Lip. (b) Relative transcript levels of *PeNAC67*, *PeSCL23* and *PeMYB4* in the lip-like petal and lip of *P. equestris* var.trilip at S8 development stage. (c) and (d) The deduced peptide sequence of PeNAC67. (c) Polypeptide alignment: The NAC domain contains ABCDE five subdomains and α-helix regions are indicated by green bars. (d) Phylogeny of PeNAC67. (e) and (f) The deduced peptide sequence of PeSCL23. (e) Polypeptide alignment: GRAS domains are color-coded (f) Phylogeny of PeSCL23 with others.

**Additional file 3: Fig. S3.** Yeast one hybrid verification for PeNAC67 binding the promoters of *MADS* genes.

**Additional file 4: Fig. S4.** Identification of TF-encoding genes expressed in orchid floral organs using RNA-seq. (a) (Upper panel) Flowers of *P. equestris* and *P. equestris* var.trilip showing different floral organs. (Lower panel) Venn diagram showing the numbers of differentially expressed genes (DEGs), with 28 TF-encoding genes identified from these common DEGs using Plant Transcription Factor Database. (b) The number of family members of 28 TF-encoding genes. (c) Summary of these 28 DEGs encoding TFs. (d) The heatmap of differential gene expression. Expression values for each gene are normalized across all samples by Z-score normalization. (e) Yeast two hybrid for PeSCL with proteins encoding candidate genes from RNA seq. (f) Yeast two hybrid for PeNAC67 with proteins encoding candidate genes from RNA seq.

**Additional file 5: Table S1.** Primer Sequences. **Table S2.** RNA-seq sequencing data statistics. **Table S3.** RNA-seq data quality control. **Table S4.** RNA-seq comparison data statistics.

## Data Availability

The data that support the findings of this study are available from the corresponding author upon reasonable request. All raw and processed data files have been deposited to the National Center for Biotechnology Information (NCBI) Sequence Read Archive (SRA) under accession number of PRJNA899518: ATAC-seq of *P. equestris* var. trilip, and BioProject: Processed PRJNA899374: RNA-seq of *P. equestris* and *P. equestris* var.trilip.

## Abbreviations

TF: Transcription factor
P*.equestris*: *Phalaenopsis equestris*
P code: Perianth code
DPI: Days post-infection
ATAC-seq: Assay for transposase accessible chromatin with high throughput sequencing
VIGS: Virus-induced gene silencing
BiFC: Bimolecular fluorescence complementarity
Co-IP: Co-immunoprecipitation
DEGs: Differentially expressed genes
PL: Lip-like petals
Li: Lips
