# Supplementary figures and images for: PeNAC67-PeKAN2-PeSCL23 and B-class MADS-box transcription factors synergistically regulate the specialization process from petal to lip in Phalaenopsis equestris
Source: Mol Hortic. 2024 Apr 23;4:15. doi: 10.1186/s43897-023-00079-8 (PMC11036780; doi:10.1186/s43897-023-00079-8)

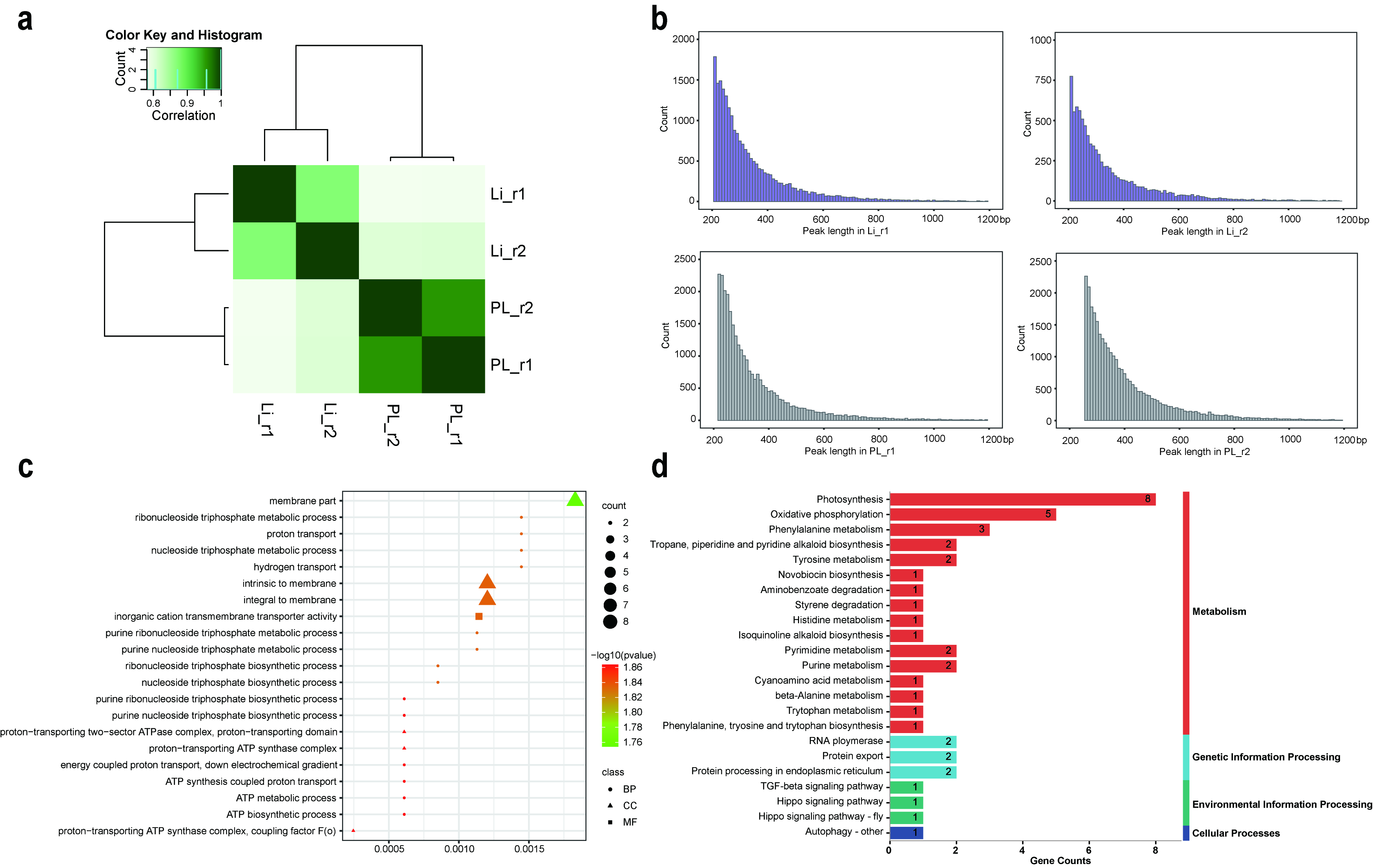

Supplement: Supplementary file 1 — Additional file 1: Fig. S1. Characteristics and functional analysis of differential peak and related gene. (a) Sample repeatability heatmap of ATAC-seq datasets. (b) Distribution of peak sizes of sequencing samples. (c) Gene Ontology annotations of differential peak-related genes. GO enrichment include three parts: Molecular Function (MF), Biological Process (BP) and Cell Component (CC). (d) KEGG pathways analysis of differential peak-related genes. [file 43897_2023_79_MOESM1_ESM.tif]

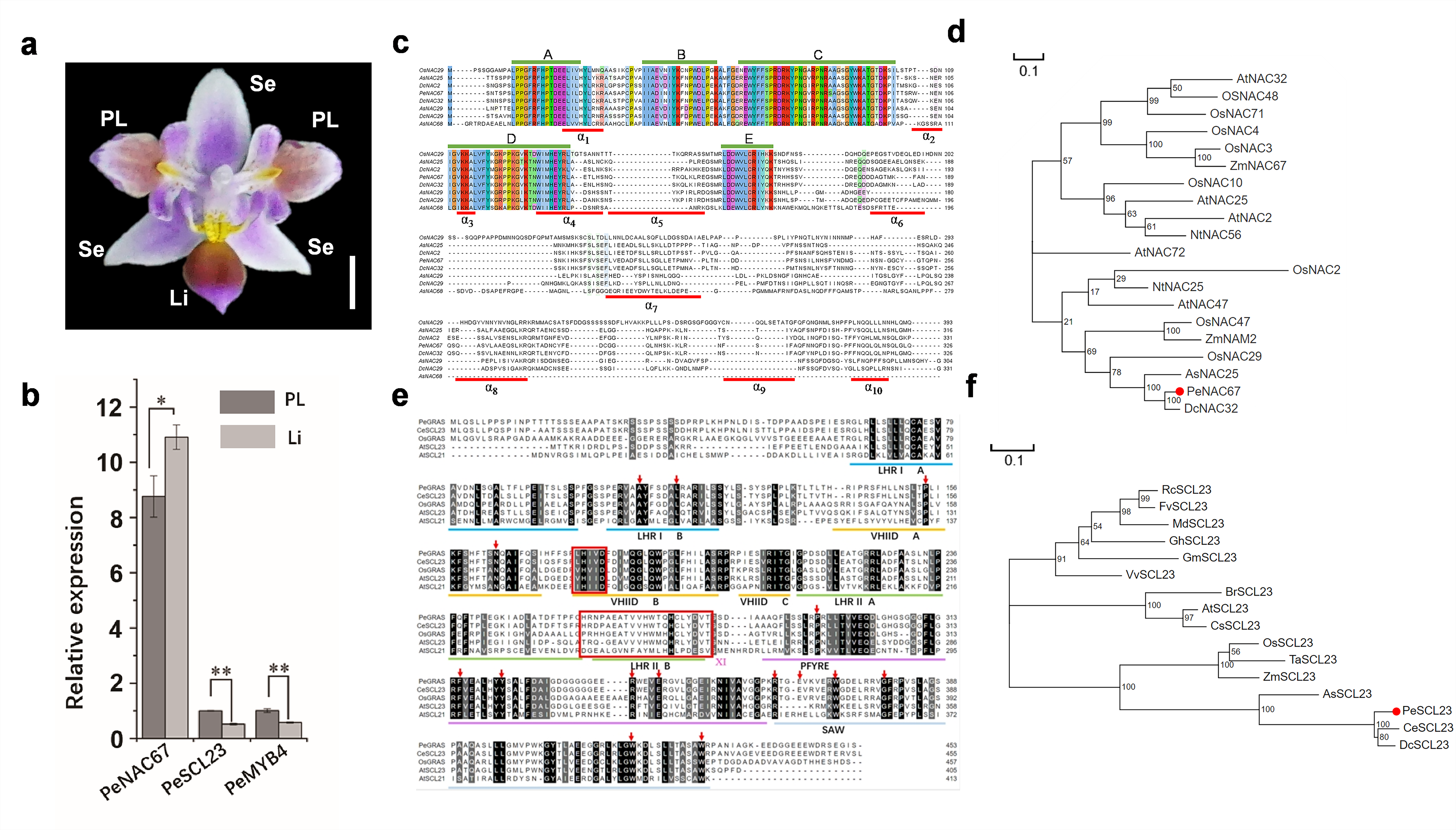

Supplement: Supplementary file 2 — Additional file 2: Fig. S2. Sequence characteristics of PeNAC67 and PeSCL23. (a) Phenotypic analysis of P. equestris var.trilip. Se: sepal, PL: lip-like petal, Li: Lip. (b) Relative transcript levels of PeNAC67, PeSCL23 and PeMYB4 in the lip-like petal and lip of P. equestris var.trilip at S8 development stage. (c) and (d) The deduced peptide sequence of PeNAC67. (c) Polypeptide alignment: The NAC domain contains ABCDE five subdomains and α-helix regions are indicated by green bars. (d) Phylogeny of PeNAC67. (e) and (f) The deduced peptide sequence of PeSCL23. (e) Polypeptide alignment: GRAS domains are color-coded (f) Phylogeny of PeSCL23 with others. [file 43897_2023_79_MOESM2_ESM.tif]

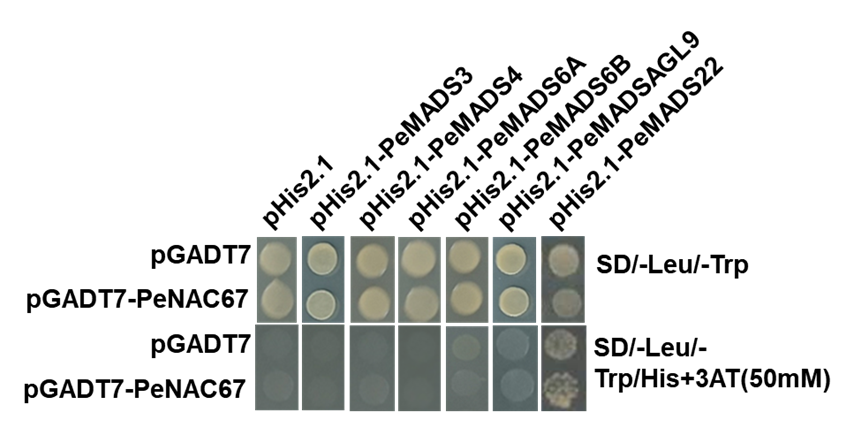

Supplement: Supplementary file 3 — Additional file 3: Fig. S3. Yeast one hybrid verification for PeNAC67 binding the promoters of MADS genes. [file 43897_2023_79_MOESM3_ESM.tif]

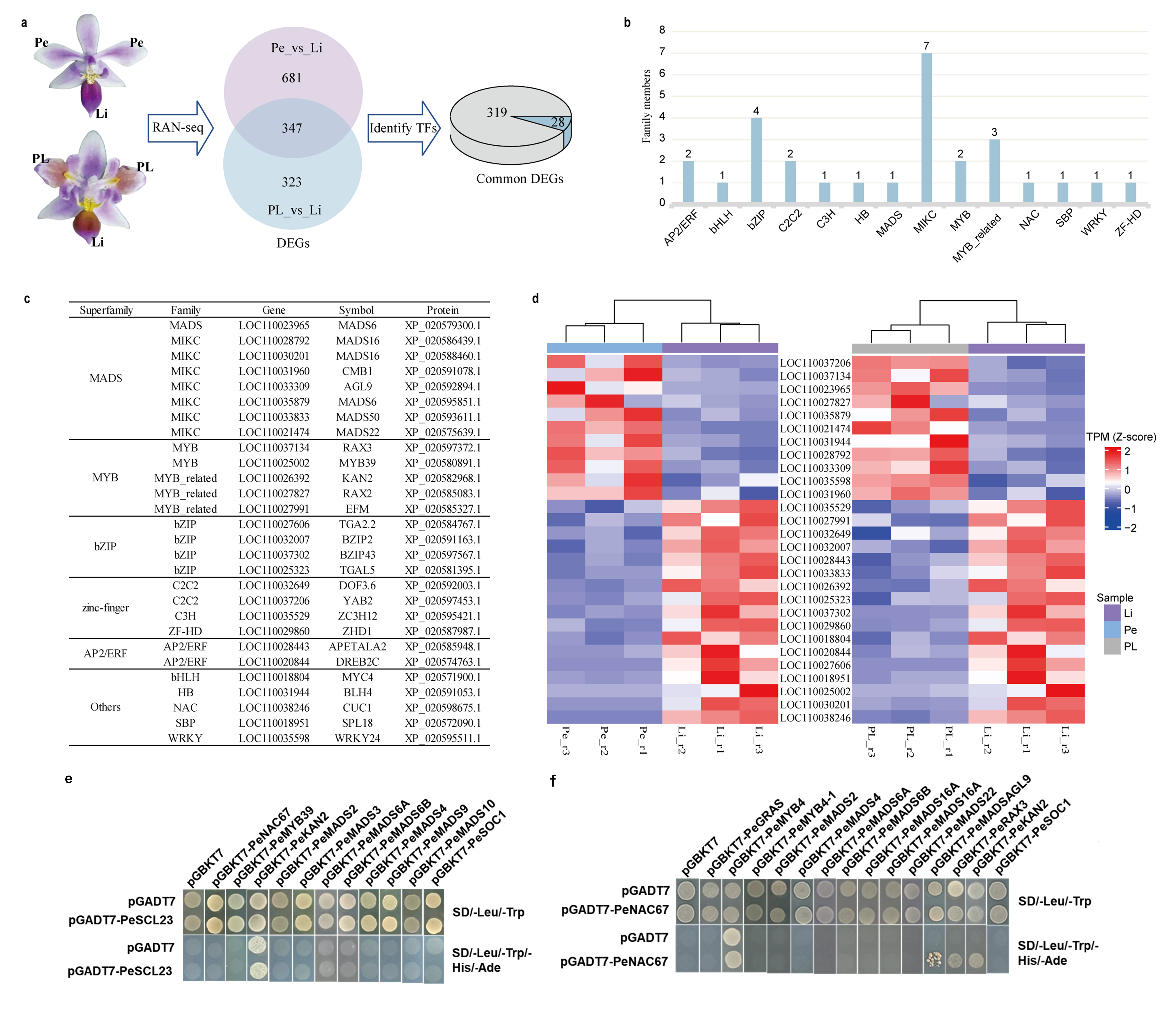

Supplement: Supplementary file 4 — Additional file 4: Fig. S4. Identification of TF-encoding genes expressed in orchid floral organs using RNA-seq. (a) (Upper panel) Flowers of P. equestris and P. equestris var.trilip showing different floral organs. (Lower panel) Venn diagram showing the numbers of differentially expressed genes (DEGs), with 28 TF-encoding genes identified from these common DEGs using Plant Transcription Factor Database. (b) The number of family members of 28 TF-encoding genes. (c) Summary of these 28 DEGs encoding TFs. (d) The heatmap of differential gene expression. Expression values for each gene are normalized across all samples by Z-score normalization. (e) Yeast two hybrid for PeSCL with proteins encoding candidate genes from RNA seq. (f) Yeast two hybrid for PeNAC67 with proteins encoding candidate genes from RNA seq. [file 43897_2023_79_MOESM4_ESM.tif]
