# Supplementary material for: PeNAC67-PeKAN2-PeSCL23 and B-class MADS-box transcription factors synergistically regulate the specialization process from petal to lip in Phalaenopsis equestris
Source: Mol Hortic. 2024 Apr 23;4:15. doi: 10.1186/s43897-023-00079-8 (PMC11036780; doi:10.1186/s43897-023-00079-8)
Supplement: Supplementary file 5 — Additional file 5: Table S1. Primer Sequences. Table S2. RNA-seq sequencing data statistics. Table S3. RNA-seq data quality control. Table S4. RNA-seq comparison data statistics. [file 43897_2023_79_MOESM5_ESM.docx]

**Table S1 Primer Sequences**

| Primer | | | Sequences |
| --- | --- | --- | --- |
| CymMV-PeNAC67-F | | | GGGGACAAGTTTGTACAAAAAAGCAGGCTGCGTGAAGACTAACTGGATCATGC |
| CymMV-PeNAC67-R | | | GGGGACCACTTTGTACAAGAAAGCTGGGTGATTGATTTGGTTGCGTGCCGTAG |
| CymMV-PeSCL23-F1 | GGGGACAAGTTTGTACAAAAAAGCAGGCTATGCTCCAAAGCCTCCTCCCTC | | |
| CymMV-PeSCL23-R1 | GGGGACCACTTTGTACAAGAAAGCTGGGTGATTGTCGACGGCGACTGATTCG | | |
| CymMV-PeSCL23-R2 | GGGGACAAGTTTGTACAAAAAAGCAGGCTCTTCCAATCCATCCACTTCTTCT | | |
| CymMV-PeSCL23-R2 | GGGGACCACTTTGTACAAGAAAGCTGGGTACGACAGTAGCTTCTGCGGGGTT | | |
| CymMV-PeKAN2-F | GGGGACAAGTTTGTACAAAAAAGCAGGCTACTCACAAGTCCCTCTCCCTCAAC | | |
| CymMV-PeKAN2-R | GGGGACCACTTTGTACAAGAAAGCTGGGTTTCATGGCCACCGAGGAGCTCAAC | | |
| qPeNAC67-F | CAGAACAAAGCCTGAAGCGA | | |
| qPeNAC67-R | GAGATGTCAAACTGGTTGTTAAAT | | |
| qPeSCL23-F | GCCGCACAATTCTTATCCT | | |
| qPeSCL23-R | CCCAATCGCATCAAACAG | | |
| qPeActin-F | GCTGAGGGAGGCAAGGATAGAT | | |
| qPeActin-R | GCACCCAGCAGCATGAAGATC | | |
| qPeKAN2-F | TTCCTCTTCCTCACCATCTC | | |
| qPeKAN2-R | AGGAGCTCAACGGCATGAAC | | |
| qPeMADS3-F | GCGAGCTCACGGTTCTCTGTGATG | | |
| qPeMADS3-R | AAATTTATGCCGGACACCTGCTGG | | |
| qPeMADS9-F | ACCTTCTCCAAGCGCAGAAATGGG | | |
| qPeMADS9-R | ATGTCAGGGCTGCCAAACTCGAAG | | |
| AD-PeNAC67-F | ATGGCCATGGAGGCCAGTGAATTCATGTCGAACACCTCACCTTCACTC | | |
| AD-PeNAC67-R | CTGCAGCTCGAGCTCGATGGATCCTCACTGCAATCCCAATTGAGAATT | | |
| BD-PeNAC67-F | CATATGGCCATGGAGGCCGAATTCATGTCGAACACCTCACCTTCACTC | | |
| BD-PeNAC67-R | CGGCCGCTGCAGGTCGACGGATCCTCACTGCAATCCCAATTGAGAATT | | |
| AD-PeKAN2-F | ATGGCCATGGAGGCCAGTGAATTCATGGAGTTGTTTTCCGCTCAACCA | | |
| AD-PeKAN2-R  BD-PeKAN2-R | CTGCAGCTCGAGCTCGATGGATCCCTATGACCTTCCCAAAGTGAAGTC  CGGCCGCTGCAGGTCGACGGATCCCTATGACCTTCCCAAAGTGAAGTC | | |
| BD-PeKAN2-F(1-942) | CATATGGCCATGGAGGCCGAATTCATGGAGTTGTTTTCCGCTCAACCA | | |
| BD-PeKAN2-R(1-942) | CGGCCGCTGCAGGTCGACGGATCCCTATGACCTTCCCAAAGTGAAGTC | | |
| BD-KAN2-F1(1-390) | CATATGGCCATGGAGGCCGAATTCATGGAGTTGTTTTCCGCTCAACC | | |
| BD-KAN2-R1(1-390) | CGGCCGCTGCAGGTCGACGGATCCCTAAAACCTTGAAGGCGAGCGTGAG | | |
| BD-KAN2-F2(391-620) | CATATGGCCATGGAGGCCGAATTCATGCTTCCCCGGTTTCCAGCAAAGA | | |
| BD-KAN2-R2(391-620) | CGGCCGCTGCAGGTCGACGGATCCCTAGATGAAGTTGCTGCCTTATCAGTGG | | |
| BD-KAN2-F3(621-942) | CATATGGCCATGGAGGCCGAATTCATGAGGACAAAGTGAGCCCATTGAG | | |
| BD-KAN2-R3(621-942) | CGGCCGCTGCAGGTCGACGGATCCCTATGACCTTCCCAAAGTGAAGTCA | | |
| AD-PeSCL23-F | ATGGCCATGGAGGCCAGTGAATTCATGCTCCAAAGCCTCCTCCCTCC | | |
| AD-PeSCL23-R | CTGCAGCTCGAGCTCGATGGATCCTCAAAAAGAGATCCCTTCACTCC | | |
| BD-PeSCL23-F | CATATGGCCATGGAGGCCGAATTCATGCTCCAAAGCCTCCTCCCTCC | | |
| BD-PeSCL23-R | CGGCCGCTGCAGGTCGACGGATCCTCAAAAAGAGATCCCTTCACTCC | | |
| BD-PeMADS2-F | CATATGGCCATGGAGGCCGAATTCATGGGGAGGGGGAAGATAGAGATA | | |
| BD-PeMADS2-R | CGGCCGCTGCAGGTCGACGGATCCTTATGCAAGGCTAAGATCATGTGATTC | | |
| BD-PeMADS3-F | CATATGGCCATGGAGGCCGAATTCATGGGGAGGGGGAAGATCGAGATA | | |
| BD-PeMADS3-R  BD-PeMADS4-F  BD-PeMADS4-R  BD-PeMADS6A-F  BD-PeMADS6A-R  BD-PeMADS6B-F  BD-PeMADS6B-R  BD-PeMADS9-F  BD-PeMADS9-R  BD-PeMADS10-F  BD-PeMADS10-R  BD-PeMADS16A-F  BD-PeMADS16A-R  BD-PeMADS22-F  BD-PeMADS22-R  BD-PeMADSAGL9-F  BD-PeMADSAGL9-R  BD-PeMYB4-F  BD-PeMYB4-R  BD-PeMYB4-1-F  BD-PeMYB4-1-R  BD-PeMYB39-F  BD-PeMYB39-R  BD-PeSOC1-F  BD-PeSOC1-R  BD-PeRAX3-F  BD-PeRAX3-R | CGGCCGCTGCAGGTCGACGGATCCTCAGGCGAGACGTAGATCATGAGG  CATATGGCCATGGAGGCCGAATTCATGGGGAGGGGGAAGATAGAGA  GGGGACCACTTTGTACAAGAAAGCTGGGTTCAGATTGCGATTGATCTCCTTCG  CATATGGCCATGGAGGCCGAATTCATGCTGAAGAGGATTGAGAACAAGATCAATC  CGGCCGCTGCAGGTCGACGGATCCTTAGAGCATCCATCCAAGCATAAAAT  CATATGGCCATGGAGGCCGAATTCATGGGGAGAGGCAGAGTAGAG  CGGCCGCTGCAGGTCGACGGATCCTCAAAGAGTCCAGCCCCGCATGAA  CATATGGCCATGGAGGCCGAATTCATGGGGAGAGGCAGAGTAGAGCT  CGGCCGCTGCAGGTCGACGGATCCTCAAAGAGTCCAGCCCCGCATGA  CATATGGCCATGGAGGCCGAATTCATGGGGAGAGGAAGAGTGGAGC  CGGCCGCTGCAGGTCGACGGATCCTTAGAGCATCCATCCAAGCATAA  CATATGGCCATGGAGGCCGAATTCATGGCCAACTATTTCCGACTGGG  CGGCCGCTGCAGGTCGACGGATCCTCAAGCGAGGCGAAGATCGT  CATATGGCCATGGAGGCCGAATTCATGGCGAGGGAGAAGATAAAGATAAGG  CGGCCGCTGCAGGTCGACGGATCCTCACTTCCAGCCTGAGCAAGA  CATATGGCCATGGAGGCCGAATTCATGGGAAGAGGGAGAGTGGAGC  CGGCCGCTGCAGGTCGACGGATCCCTACTCGTAGGAGCCTGAAATTTGTCC  CATATGGCCATGGAGGCCGAATTCATGGTGAGAGCTCCATGCTGC  CGGCCGCTGCAGGTCGACGGATCCTCAAATCTGTGGAAATTCTTCAGCTTG  CATATGGCCATGGAGGCCGAATTCATGGTGAGAGCTCCTTGCTGT  CGGCCGCTGCAGGTCGACGGATCCTTACAAATTTCCAGATTCAGCCAACA  CATATGGCCATGGAGGCCGAATTCATGGGAAGATCTCCTTGTTGTGAC  CGGCCGCTGCAGGTCGACGGATCCTCAAGCCAATTCATTCATGAATGAATCAT  CATATGGCCATGGAGGCCGAATTCATGGTGAGGGGAAGGACGG  CGGCCGCTGCAGGTCGACGGATCCTCATCCTTTCAACAACTGTTGGGTTC  CATATGGCCATGGAGGCCGAATTCATGGGAAGAGCTCCATGCTGC  CGGCCGCTGCAGGTCGACGGATCCGCGGCAATCAAAGACCACCTG | | |
| PXY106-PeSCL23-F | ATCGAGGACGCCGGCGGATCCATGCTCCAAAGCCTCCTCCCTC | | |
| PXY106-PeSCL23-R | ACGAAAGCTCTGCAGGTCGACTCAAAAAGAGATCCCTTCACTCC | | |
| PXY104-PeKAN2-F | ATTACAGGTACCCGGGGATCCATGGAGTTGTTTTCCGCTCAACC | | |
| PXY104-PeKAN2-R | CACGCTGCCACCGCCGTCGACTGACCTTCCCAAAGTGAAGTCAA | | |
| QBV3-PeNAC67-F | AAAAAAGCAGGCTCAGGGGATATC ATGTCGAACACCTCACCTTCACTC | | |
| QBV3-PeNAC67-R | GAAAGCTGGGTGCAGGGCGATATC CTGCAATCCCAATTGAGAATTCAG | | |
| QBV3-PeNAC67-Flag-R | GTCTTTGTAGTCCTCGACGATATCCTGCAATCCCAATTGAGAATTCAG | | |
| QBV3-PeSCL23-F | AAAAAAGCAGGCTCAGGGGATATCATGCTCCAAAGCCTCCTCCCTCC | | |
| QBV3-PeSCL23-HA-R | GGTAATTGTAAATGTAATGATATCAAAAGAGATCCCTTCACTCCTGTC | | |
| QBV3-PeSCL23-Myc-R | ACCGTTAATTAACCCGCTGATATCAAAAGAGATCCCTTCACTCC | | |
| QBV3-PeKAN2-F | AAAAAAGCAGGCTCAGGGGATATCATGGAGTTGTTTTCCGCTCAACC | | |
| QBV3-PeKAN2-R | GAAAGCTGGGTGCAGGGCGATATCTGACCTTCCCAAAGTGAAGTC | | |
| QBV3-PeKN2-HA-R | GGTAATTGTAAATGTAATGATATCTGACCTTCCCAAAGTGAAGT | | |
| QBV3-PeKAN2-Myc-R | ACCGTTAATTAACCCGCTGATATCTGACCTTCCCAAAGTGAAGTCAAG | | |
| QBV3-PeMADS3-F | AAAAAAGCAGGCTCAGGGGATATCATGGGGAGGGGGAAGATCGAGATA | | |
| QBV3-PeMADS3-R | | GAAAGCTGGGTGCAGGGCGATATCGGCGAGACGTAGATCATGAGGGAC | |
| QBV3-PeMADS3-FLAG-R | | GTCTTTGTAGTCCTCGACGATATCGGCGAGACGTAGATCATGAGGGAC | |
| QBV3-PeMADS9-F | AAAAAAGCAGGCTCAGGGGATATCATGGGGAGAGGCAGAGTAGAGCT | | |
| QBV3-PeMADS9-R | GAAAGCTGGGTGCAGGGCGATATCAAGAGTCCAGCCCCGCATGA | | |

**Table S2 RNA-seq sequencing data statistics**

| Sample | Raw reads | Clean reads | Error rate(%) | Q20(%) | Q30(%) | GC content (%) |
| --- | --- | --- | --- | --- | --- | --- |
| Li_1 | 35901564 | 35900174 | 0.0246 | 98.22 | 94.49 | 48.28 |
| Li_2 | 38319102 | 38317764 | 0.0243 | 98.32 | 94.76 | 48.21 |
| Li_3 | 35376386 | 35375022 | 0.0244 | 98.3 | 94.7 | 48.12 |
| Pe_1 | 32628804 | 32627892 | 0.0245 | 98.27 | 94.62 | 48.17 |
| Pe_2 | 27069864 | 27068770 | 0.0245 | 98.22 | 94.66 | 48.98 |
| Pe_3 | 21718994 | 21718360 | 0.0247 | 98.14 | 94.47 | 48.42 |
| PL_1 | 30319440 | 30318556 | 0.0246 | 98.23 | 94.55 | 48.08 |
| PL_2 | 23584698 | 23584002 | 0.0241 | 98.39 | 94.99 | 48.38 |
| PL_3 | 25810066 | 25808806 | 0.025 | 98.02 | 94.11 | 48.23 |

**Table S3 RNA-seq data quality control**

| Sample | Clean reads | Clean bases | Error rate(%) | Q20(%) | Q30(%) | GC content (%) |
| --- | --- | --- | --- | --- | --- | --- |
| Li_1 | 35900174 | 5359565060 | 0.0246 | 98.22 | 94.49 | 48.28 |
| Li_2 | 38317764 | 5717196845 | 0.0243 | 98.32 | 94.76 | 48.21 |
| Li_3 | 35375022 | 5290566345 | 0.0244 | 98.3 | 94.7 | 48.12 |
| Pe_1 | 32627892 | 4887017898 | 0.0245 | 98.27 | 94.62 | 48.17 |
| Pe_2 | 27068770 | 4046149496 | 0.0245 | 98.22 | 94.66 | 48.98 |
| Pe_3 | 21718360 | 3253769674 | 0.0247 | 98.14 | 94.47 | 48.42 |
| PL_1 | 30318556 | 4509874393 | 0.0246 | 98.23 | 94.55 | 48.08 |
| PL_2 | 23584002 | 3527893068 | 0.0241 | 98.39 | 94.99 | 48.38 |
| PL_3 | 25808806 | 3869151562 | 0.025 | 98.02 | 94.11 | 48.23 |

**Table S4 RNA-seq comparison data statistics**

| Sample | Total reads | Total mapped | Multiple mapped | Uniquely mapped |  |
| --- | --- | --- | --- | --- | --- |
| Li_1 | 35900174 | 23992558(66.83%) | 1071130(2.98%) | 22921428(63.85%) |  |
| Li_2 | 38317764 | 27353207(71.39%) | 1179743(3.08%) | 26173464(68.31%) |  |
| Li_3 | 35375022 | 23767323(67.19%) | 1050837(2.97%) | 22716486(64.22%) |  |
| Pe_1 | 32627892 | 18141880(55.6%) | 827773(2.54%) | 17314107(53.07%) |  |
| Pe_2 | 27068770 | 11376151(42.03%) | 552618(2.04%) | 10823533(39.99%) |  |
| Pe_3 | 21718360 | 11194651(51.54%) | 521082(2.4%) | 10673569(49.15%) |  |
| PL_1 | 30318556 | 20261985(66.83%) | 861107(2.84%) | 19400878(63.99%) |  |
| PL_2 | 23584002 | 11362198(48.18%) | 507261(2.15%) | 10854937(46.03%) |  |
| PL_3 | 25808806 | 14510925(56.22%) | 634402(2.46%) | 13876523(53.77%) |  |
